# Comprehensive analysis of metabolome and transcriptome reveals the mechanism of color formation in different leave of *Loropetalum Chinense* var. *Rubrum*

**DOI:** 10.1186/s12870-023-04143-9

**Published:** 2023-03-08

**Authors:** Xia Zhang, Li Zhang, Damao Zhang, Dingding Su, Weidong Li, Xiangfei Wang, Qianru Chen, Wenqi Cai, Lu Xu, Fuxiang Cao, Dongling Zhang, Xiaoying Yu, Yanlin Li

**Affiliations:** 1 Institute of Vegetables and Flowers, Chinese Academy of Agricultural Sciences, 100081 Beijing, China; 2 College of Horticulture, Hunan Agricultural University, 410128 Changsha, China; 3 Engineering Research Center for Horticultural Crop Germplasm Creation and New Variety Breeding, Ministry of Education, 410128 Changsha, China; 4 Hunan Mid-subtropical Quality Plant Breeding and Utilization Engineering Technology Research Center, 410128 Changsha, China; 5 Hunan Horticulture Research Institute, Hunan Academy of Agricultural Sciences, 410125 Changsha, China; 6 Institute of Advanced Agricultural Sciences, Peking University, 262041 Weifang, China; 7 Hunan Key Laboratory of Innovation and Comprehensive Utilization, 410128 Changsha, China; 8 Department of Horticulture, University of Georgia, 30602 Athens, GA USA

**Keywords:** Leaf colour, Pigmentation, Anthocyanin, Synthesis pathway, *Loropetalum chinense* var. *Rubrum*

## Abstract

**Background:**

*Loropetalum chinense* var. *rubrum (L. chinense* var. *rubrum)* is a precious, coloured-leaf native ornamental plant in the Hunan Province. We found an *L. chinense* var. *rubrum* tree with three different leaf colours: GL (green leaf), ML (mosaic leaf), and PL (purple leaf). The mechanism of leaf coloration in this plant is still unclear. Therefore, this study aimed to identify the metabolites and genes involved in determining the colour composition of *L. chinense* var. *rubrum* leaves, using phenotypic/anatomic observations, pigment content detection, and comparative metabolomics and transcriptomics.

**Results:**

We observed that the mesophyll cells in PL were purple, while those in GL were green and those in ML were a mix of purple-green. The contents of chlorophyll a, b, carotenoids, and total chlorophyll in PL and ML were significantly lower than those in GL. While the anthocyanin content in PL and ML was significantly higher than that in GL. The metabolomics results showed the differences in the content of cyanidin 3-*O*-glucoside, delphinidin 3-*O*-glucoside, cyanidin 3,5-*O*-diglucoside, pelargonidin, and petunidin 3,5-diglucoside in ML, GL, and PL were significant. Considering that the change trend of anthocyanin content change was consistent with the leaf colour difference, we speculated that these compounds might influence the colour of *L. chinense* var. *rubrum* leaves. Using transcriptomics, we finally identified nine differentially expressed structural genes (one *ANR* (*ANR1217*); four *CYP75A*s (*CYP75A*1815, *CYP75A*2846, *CYP75A*2909, and *CYP75A*1716); four *UFGT*s (*UFGT*1876, *UFGT*1649, *UFGT*1839, and *UFGT*3273) and nine transcription factors (two *MYB*s (*MYB*1057 and *MYB*1211), one *MADS-box* (*MADS*1235), two *AP2-likes* (*AP2-like*1779 and *AP2-like2*234), one *bZIP* (*bZIP*3720), two *WD*40s (*WD*2173 and *WD*1867) and one *bHLH* (*bHLH*1631) that might be related to flavonoid biosynthesis and then impacted the appearance of colour in *L. chinense* var. *rubrum* leaves.

**Conclusion:**

This study revealed potential molecular mechanisms associated with leaf coloration in *L. chinense* var. *rubrum* by analyzing differential metabolites and genes related to the anthocyanin biosynthesis pathway. It also provided a reference for research on leaf colour variation in other ornamental plants.

**Supplementary Information:**

The online version contains supplementary material available at 10.1186/s12870-023-04143-9.

## Introduction

*Loropetalum chinense* var. *rubrum* belongs to the Hamamelidaceae (witch-hazel family) [1] and is mainly distributed in the belt south of the middle Yangtze River to the north of the Tropic of Cancer in China. It originated in the Hunan Province and played an important role in the landscape [2]. *L. chinense* var. *rubrum* is an evergreen plant with an elegant tree shape and brightly coloured foliage. Its leaves, flowers, and roots were used in traditional Chinese medicine for treating cough, burns, abdominal pain, etc. [3]. Recently, the plant has gained a lot of interest and has been widely cultivated for its ornamental and medicinal value.

As an ornamental plant, the colour of *L. chinense* var. *rubrum* leaves are one of its most significant characteristics. Leaf coloration is determined by the pigment in the mesophyll cells, such as chlorophyll, carotenoids, and flavonoids [4, 5]. Flavonoids are comprised of chalcones, flavone, flavonol, and anthocyanins, which colour plants blue, pink, yellow, purple, and red [6, 7]. Anthocyanins are considered to be the main coloration pigments, while flavone and flavonol are the synergistic pigments [8, 9]. Anthocyanins have been proved to determine the appearance of colour in many fruits, flowers, and vegetables, such as *Vaccinium corymbosum* [10], *Morella rubra* [11], *Centaurea cyanus* [12], *Primula vulgaris* [13], and *Allium cepa L* (Onion) [14]. In addition, they also played a vital role in the physiological activities of plants and human health. They have been shown to alleviate the stress of cold, drought, and pests on plants [15–17], and also contributed to protecting the human body from oxidative stress, cancer, bacterial infection, and cardiovascular and neurodegenerative diseases [18, 19].

**Fig. 1:**
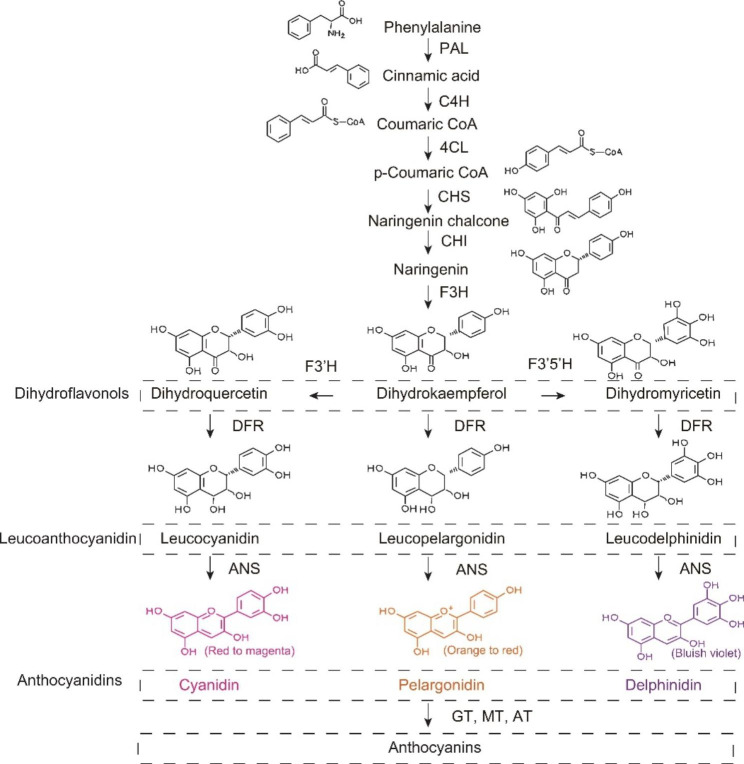
Biosynthesis pathway of anthocyanin. PAL (phenylalanine ammonia-lyase); C4H (cinnamate 4-monooxygenase); 4CL (4-coumarate-CoA ligase); CHS (chalcone synthase); CHI (chalcone isomerase); F3H (flavanone 3-hydroxylase); F3’H (flavonoid 3’-hydroxylase); F3’5’H (flavonoid 3’,5’-hydroxylase); DFR (dihydroflflavonol 4-reductase); ANS (anthocyanidin synthase); GT (glucosyltransferases); MT (methyltransferases) and AT (acyltransferases)

Anthocyanins, which are the product of the phenylpropanoid pathway, are the central issue in the study of plant colour, which is [20], and its synthetic pathway has been well-characterized in *Arabidopsis thaliana* and *Petunia* [21, 22]. First, phenylalanine is required as a substrate to be converted to cinnamic acid in the presence of phenylalanine ammonia-lyase (PAL) [23]. Cinnamic acid is then converted into various dihydroflavonols by a series of enzymes, such as cinnamate 4-hydroxylase (C4H), 4-coumarate-CoA ligase (4CL), chalcone synthase (CHS), chalcone isomerase (CHI), clavanone3-hydroxylase (F3H), flavonoid 3’-hydroxylase (F3’H) and flavonoid 3’5’ -hydroxylase (F3’5’H) [20, 24]. Subsequently, dihydroflavonol4-reductase (DFR) catalyses the conversion of dihydroflavonols to leucoanthocyanidins [25], which are finally converted into anthocyanins by anthocyanidin synthase (ANS) [26]. Anthocyanins are the end products of the anthocyanin synthesis pathway and are divided into six groups: cyanidin, pelargonidin, delphinidin, peonidin, petunidin, and malvidin [27, 28]. However, they are unstable in the cytoplasm and require further glycosylation (GT), methylation (MT), and acylation (AT) to be stored in vacuoles [29] (Fig. 1). The anthocyanin derivatives produced vary among plant species. For example, anthocyanin 3-*O*-glucoside in *Ipomoea nil* was glycosylated to form anthocyanin 3-*O*-sophoroside [30]; *Vitis vinifera* was pigmented through glycosylation and methylation to generate procyanidin-3-glucoside and paeoniflorin-3-glucoside [31]; *Chrysanthemum×morifolium* was pigmented by acylating cyanidin 3-*O*-glucoside to cyanidin-3-*O*-(6’’-malonylglucoside) [32].

The transcription factor families *MYB*, *bHLH*, and *WD*40 are also involved in anthocyanin synthesis by regulating the expression of structural genes [33]. *MYB* is one of the most abundant family of transcription factors in higher plants, which is related to regulating secondary metabolism, cell morphogenesis and differentiation, signal transduction, and stress response [34–37]. They regulate the expression of the early genes in anthocyanin synthesis [38], such as *PAL*, *C4H*, *4CL*, *CHS*, and *CHI* [39–41]. The *bHLH* transcription factors regulate anthocyanin synthesis by binding to either *MYB* transcription factors or WD40 proteins, or both of them to form an MBW protein complex [42–44]. Therefore, a thorough investigation is warranted to find out the relationship between leaf colour and anthocyanin species.

In this study, the transcriptional and metabolic data on *L. chinense var*. *rubrum* leaves of three colours (green, mosaic and purple) were compared to identify the key metabolites and genes that regulate leaf colour formation, to clarify the molecular and metabolic mechanisms underlying the different pigmentations and to provide a basis for colour improvement in ornamental plants.

## Results

## Leaf colour observation and pigment content determination

**Fig. 2:**
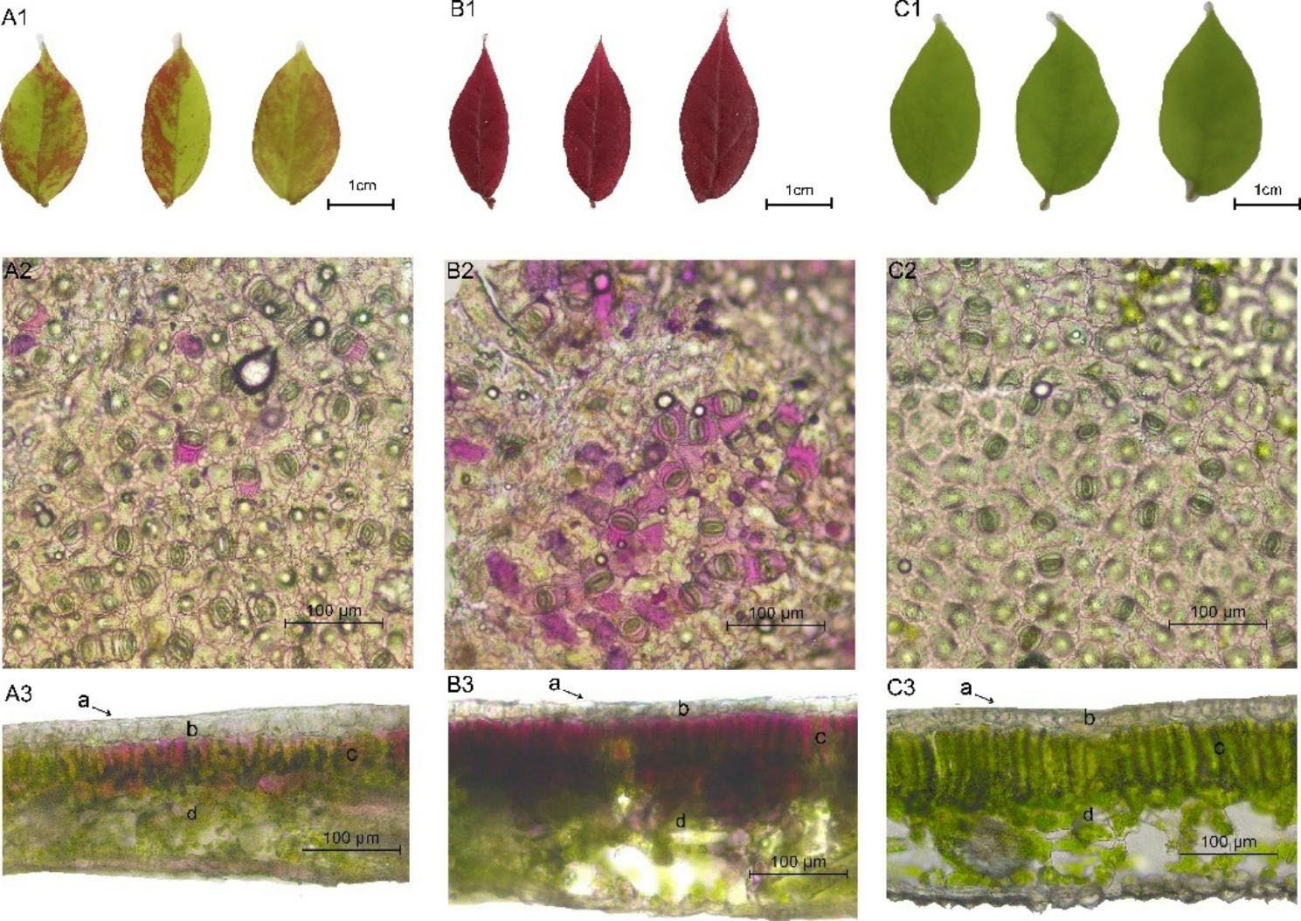
Pictures show mesophyll cells and sections of ML, PL, and GL leaves in turn, scale bars = 100 μm. **A1-C1** ML, PL, and GL leaves; **A2-C2** Microscopic observation of leaf epithelial cell, scale bars = 100 μm; **A3-C3** The anatomical structure of a transverse section of the blade, scale bars = 100 μm

To understand the general colour characteristics of *L. chinense* var. *rubrum* leaves, leaves of three colours were observed quantitatively, anatomically, and microscopically. The colour of the leaves was consistent with that of the pigment cells or cell clusters inside. The three colours of leaves that were examined were: green leaves (GL), purple leaves (PL), and mosaic leaves (ML) (Fig. 2A1-C1). When observed at a magnification of 20×, the upper epidermal cells of ML leaves had a small amount of purple pigment, while those of PL leaves had a large amount of purple pigment, and those of GL leaves had a large amount of green pigment (Fig. 2A2-C2). Meanwhile, the transverse section was observed to show that the *L. chinense var*. *rubrum* leaves had typical structural characteristics such as upper epidermis, palisade tissue, sponge tissue, and lower epidermis (Fig. 2A3-C3). Chlorophyll and anthocyanin were mainly in the mesophyll cells of leaves. The mesophyll cells of ML leaves were purple and green, while those of PL leaves were mostly purple and those of GL leaves were green ( Fig. 2A3-C3).

To further evaluate the leaf colour objectively, we used the CIELAB system to detail various leaf colour indexes (L*, a*, b*) and detect pigment contents. The L* (lightness) parameter varies from 100 (white) to 0 (black), A positive value of a* indicates more red than green, and a positive value of b* means more yellow than blue [45]. Chromatic value analysis showed that the L* and b* of GL leaves were significantly higher than those of ML leaves, while the a* value of ML and PL leaves was higher compared with GL leaves (Table 1).

**Fig. 3:**
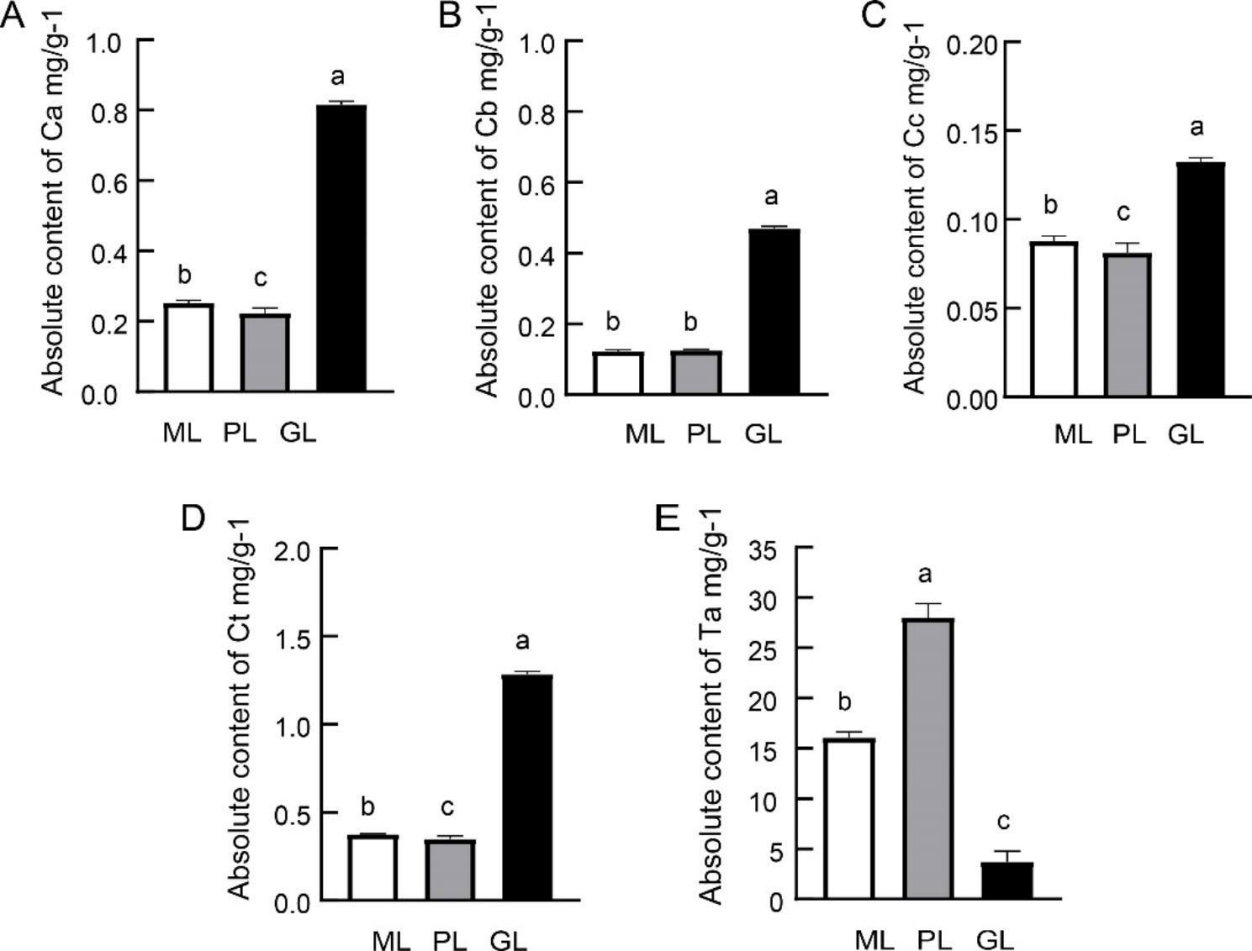
The X-axis indicates the name of the sample, and the Y-axis indicates the absolute content of the extracted fresh weight. **A** Absolute content of chlorophyll (A) **B** Absolute content of chlorophyll (B) **C** Absolute content of carotenoid concentration. **D** Absolute contents of total chlorophyll. **E** Absolute contents of total anthocyanins. The data represent six biological repeats and their average. Mean (± SE) with different lower letters are significantly different within the (mean separation by LSD and Duncan’s test at *P* < 0.05)

We quantified the photosynthetic pigment and total anthocyanin contents in the leaves of the three samples (Fig. 3) (Additional file 1). For photosynthetic pigment, we found notable differences among the three samples. GL had the highest content of photosynthetic pigment, around 2-3-fold higher than ML and PL, while the corresponding differences between ML and PL were insignificant (Fig. 3A, B, C, D). PL had the highest anthocyanin content (Fig. 3E).

**Table 1:** The leaf color difference values of ML, PL, and GL.

Materials	RHSCC values	L*	a*	b*	Color index
ML	144B and 183 A	63.26 ± 1.11b	-1.13 ± 0.75b	6.56 ± 2.57b	2.60 ± 0.11a
PL	N77A	60.48 ± 0.38c	8.72 ± 0.58a	-4.79 ± 0.23c	2.56 ± 0.02a
GL	144 A	68.59 ± 0.38a	-13.44 ± 0.77c	24.65 ± 2.28a	1.87 ± 0.05b

Note: ML, Mosaic leaves; Purple leaves; GL, Green Leaves; L*, lightness; a*, red/green value; and b* blue/yellow value; Color index. Each sample was detected with three biological repetitions. Mean (± SE) with different lower letters are significantly different within the (mean separation by LSD and Duncan’s test at *P* < 0.05)

### Note

a-cuticle; b-epidermis from adaxial leaf surface; c-palisade issue; d-spongy issue.

## Statistical analysis of metabolomic data

**Fig. 4:**
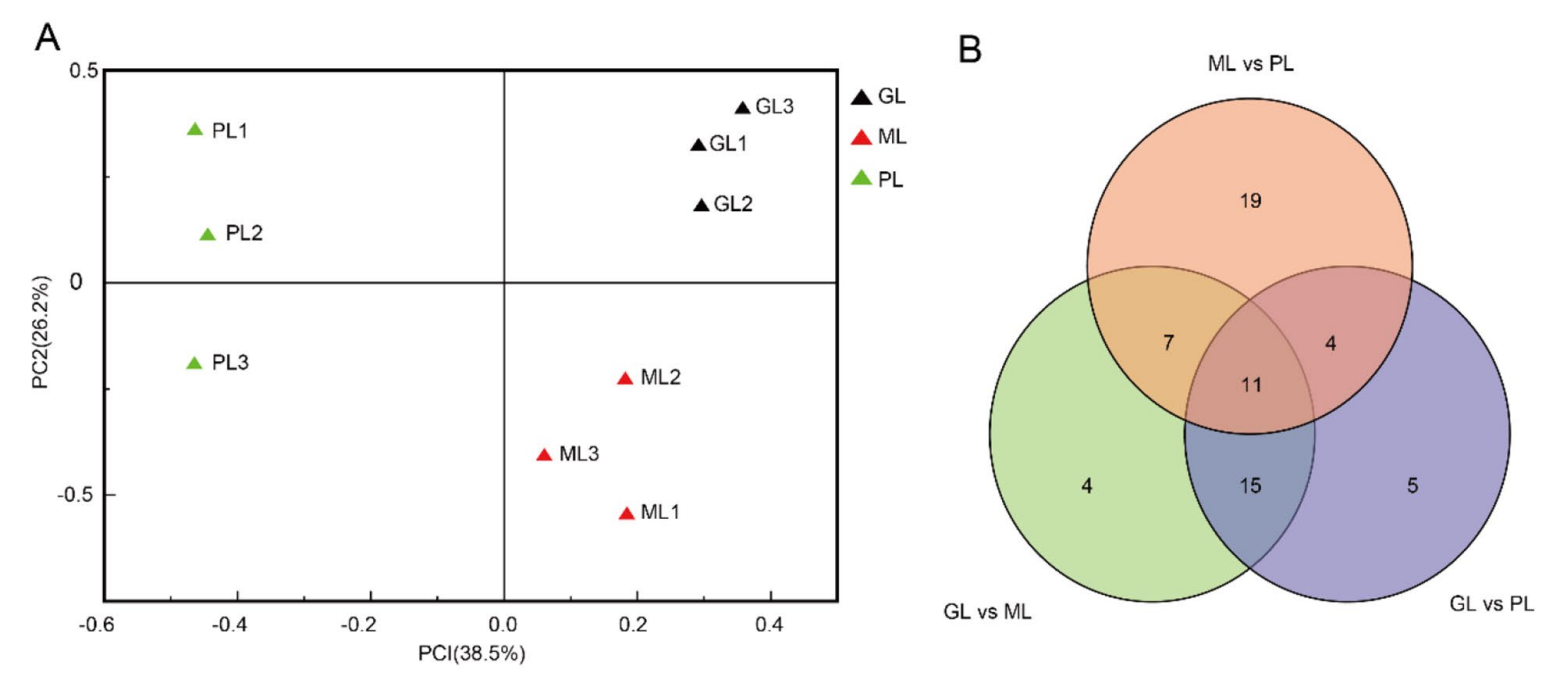
Differential metabolites from different leaves. **A** PCA score plot of three materials and numbers of potential markers for each leaf color. **B** Venn diagram shows the overlapping and cultivar-specific differential metabolites from ML, PL, and GL

Physiological data showed that the anthocyanin contents in the different-coloured leaves of *L. chinense* var. *rubrum* differed significantly; however, the reason for this difference remains unclear. We profiled the metabolome of the three samples using the liquid chromatography-tandem mass spectrometry metabolomics approach. A total of 207 compounds were detected in the *L. chinense* var. *rubrum* leaf, which was grouped into eight classes: proanthocyanins, polyphenol, isoflavone, flavonol, flavanone, anthocyanins, flavonoids, and flavones (Additional file 2). The 207 metabolites were analyzed by principal component analysis to compare the metabolite compositions involved in the pigmentation of the leaves. The compositions of the three samples separated significantly in the first principal component (38.5% of the total variable) and the second principal component (26.2% of the total variable) (Fig. 4A), indicating that the ML, PL, and GL leaves had significant inter-group differences.

A total of 37, 35, and 41 differential metabolites were selected in GL vs. ML, GL vs. PL, and ML vs. PL, respectively (Additional file 3), with a total of 11 overlaps (Fig. 4B). The annotations of the different groups of metabolites in the various pathways of flavonoid biosynthesis (ko0094) and anthocyanin biosynthesis (ko00942) were shown in Additional file 4.

## Anthocyanin content in the three-coloured leaves

Interestingly, the leaf colours are closely related to the content of anthocyanin-related metabolites. Using metabolomics, we isolated and identified 15 anthocyanins from leaf extracts cyanidin 3-*O*-glucoside, peonidin, cyanidin *O*-syringic acid, delphinidin, delphinidin 3-*O*-glucoside, cyanidin 3-*O*-rutinoside, cyanidin 3,5-*O*-diglucoside, pelargonin, petunidin 3-*O*-glucoside, pelargonidin 3-*O*-beta-*D*-glucoside, cyanidin, cyanidin 3-*O*-galactoside, petunidin 3,5-diglucoside, malvidin 3-acetyl-5-diglucoside, and peonidin 3-sophoroside-5-glucoside. Notably, the results of total anthocyanin content were consistent with those of anthocyanin content determination, following the trend PL > ML > GL leaves (Fig. 2E). The same kind of anthocyanins was examined from the three-coloured leaves, but their levels differed significantly (Table 2). The leaves of different colours might differ in anthocyanin biosynthesis or the expression of regulatory genes.

**Table 2:** Type and content of anthocyanins in leaves of ML, PL, and GL.

Metabolites	Peak area		
	Mosaic leaf	Purple leaf	Green leaf
Cyanidin 3-*O*-glucoside	1.09	1.12	1.00
Peonidin	1.00	1.00	1.00
Cyanidin *O*-syringic acid	1.15	1.28	1.00
Delphinidin	1.01	0.98	1.00
Delphinidin 3-*O*-glucoside	1.17	1.23	1.00
Cyanidin 3-*O*-rutinoside	1.00	1.00	1.00
Cyanidin 3,5-*O*-diglucoside	1.22	1.29	1.00
Pelargonin	1.23	1.33	1.00
Petunidin 3-*O*-glucoside	1.00	5.91	1.00
Pelargonidin 3-*O*-beta-*D*-glucoside	0.18	1.34	1.00
Cyanidin	1.08	1.04	1.00
Cyanidin 3-*O*-galactoside	1.13	1.20	1.00
Petunidin 3,5-diglucoside	1.10	1.14	1.00
Malvidin 3-acetyl-5-diglucoside	3.81	3.94	1.00
Peonidin 3-sophoroside-5-glucoside	3.65	4.51	1.00

Note: The data above was the average of three biological replicates. Besides, each data was normalized by Log 10 functions and compared with GL.

## Sample quality control (QC) analysis

**Fig. 5:**
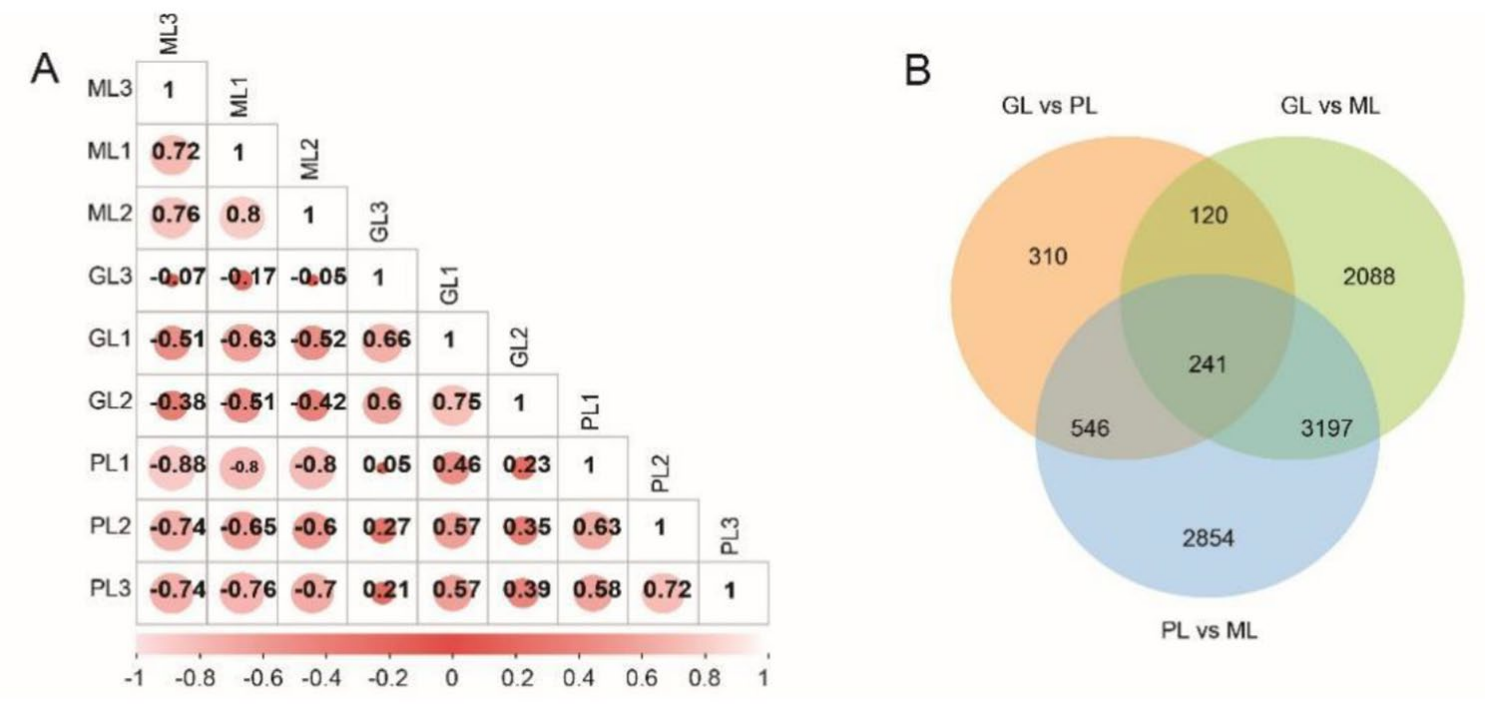
Differential expression genes in different colors. **A** Thermal diagram of the correlation coefficient between leaves. The Pearson correlation coefficient is within [-1, 1], and the closer it is to 1 or -1, the stronger the positive/anti-linear relationship. **B** Venn diagram of DEGs

Three standardized cDNA libraries were constructed from the RNA of GL, ML, and PL. After the cDNA library was cleaned and characterized, a total of 186,694,570,149,946,386 and 123,143,062 reads were obtained, respectively. The percentages of reads having Q20 (an error probability of 0.02%) were 98.09%, 97.48%, and 97.9% for GL, ML, and PL, respectively. The GC contents of the reads were approximately 43.73%, 43.98%, and 43.84%, respectively (Additional file 5). These clean reads were assembled into 231,810 unigenes ranging from 65 to 2135 bp in length (average 1271 bp) and an N50 of 2608 bp (Additional file 6). The sequencing quality covered the majority of expressed genes in GL, ML, and PL, providing a reference for further analysis. We compared the obtained sequences with the information in seven databases; 118,518 (17.88%) unigenes had homologues in the nr database, 96,572 (14.57%) in SwissProt, 115,058 (17.36%) in KEGG, 75,254 (11.36%) in KOG, 84,662 (12.77%) in GO, 88,001 (13.28%) in NT and 84,662 (12.77%) in Pfam databases, respectively (Additional file 7). We calculated the correlation coefficient of samples according to the fragments per kilobase of transcript per million mapped reads (FPKM) value to evaluate the reliability of the measured gene expression levels. The higher the similarity was, the closer the Pearson coefficient was to 1, indicating that the measurement was reliable (Fig. 5A).

## The intersection of differentially expressed genes (DEGs) in three-coloured leaves

The Venn diagram more intuitively showed the overlap of DEGs in the three comparison groups (Fig. 5B). There were 5646 DEGs (3447 downregulated, 2199 upregulated) between the GL vs. ML group, 4217 DEGs (3539 downregulated, 678 upregulated) between the GL vs. PL group and 6836 DEGs (2613 downregulated, 4223 upregulated) between the ML vs. PL group (Additional file 8).

The results of GO database annotation, presented in Additional file 9, showed that DEGs in the three-coloured can be successfully annotated into three biological processes.

The KEGG database is pathway-related. To further study the biochemical pathways of these DEGs, they were mapped onto the KEGG database [46]. Notably, KEGG pathway enrichment analysis in the pairwise comparisons of DEGs between two groups highlighted several metabolic processes including flavone and flavanol biosynthesis (ko00942) and flavonoid biosynthesis (ko00941), which were ​closely related to anthocyanin synthesis (Additional file 10). These pathways provided insights into the metabolic processes underlying different leaf pigmentations in *L. chinense* var. *rubrum*.

## Genes involved in anthocyanin biosynthesis

To further study the determinants of colour diversity in ML, PL, and GL, the anthocyanin synthesis metabolic pathway was emphasized. Anthocyanins played an important role in plant coloration. Therefore, pathways related to anthocyanin synthesis were screened out from 241 DEGs (Fig. 4B), among these nine DEGs showed significant changes in expression levels: one *ANR* (*ANR*1217), four *CYP75A* (*CYP75A*1815, *CYP75A*2846, *CYP75A*2909, and *CYP75A*1716) and four *UFGT*s (*UFGT*1876, *UFGT*1649, *UFGT1*839, and *UFGT*3273) (Additional file 11). As the key gene in the biosynthesis of delphinidin, *CYP75A* catalysed the conversion of its major substrate, dihydrokaempferol, to dihydromyricetin [47]. *ANR* [48] and *UFGT* [49] convert the substrate of anthocyanin into (-)- epicatechin and anthocyanin, respectively. In this study, we found that these nine genes (particularly *UFGT*s) were expressed considerably higher in PL than in ML and GL (Additional file 12). However, little is known about the *UFGT* gene of *L. chinense* var. *rubrum* and its function needs to be further studied.

## Genes encoding transcription factors

Transcription factors participate in the synthesis and accumulation of metabolites by modulating the expression levels of structural genes. Therefore, we screened genes related to the flavonoid synthesis pathway by comparing transcription factors between the three-coloured leaves. Genes involved in flavonoid biosynthesis are usually regulated by *MYB*, *bHLH*, *WD*40, *bZIP*, and *MADS-box* transcription factors [50]. Nine transcription factors were selected from these families (Additional file 12), including two *MYB*s (*MYB*1057 and *MYB*1211), one *MADS-box* (*MADS*1235), two *AP2-likes* (*AP2-lik*e1779 and *AP2-like*2234), one *bZIP* (*bZIP*3720), two *WD40*s (*WD*2173 and *WD*1867) and one *bHLH* (*bHLH*1631). In this study, we found that *AP2-like*2224, *bZIP*3720, *WD*1867, *WD*2173, and *bHLH*1631 were all upregulated in PL, while the *MYBs*, *MADS-box*, and *AP2-like*1779 were upregulated in ML and GL.

## Validation of transcriptome results by quantitative reverse transcription PCR (qRT-PCR)

**Fig. 6:**
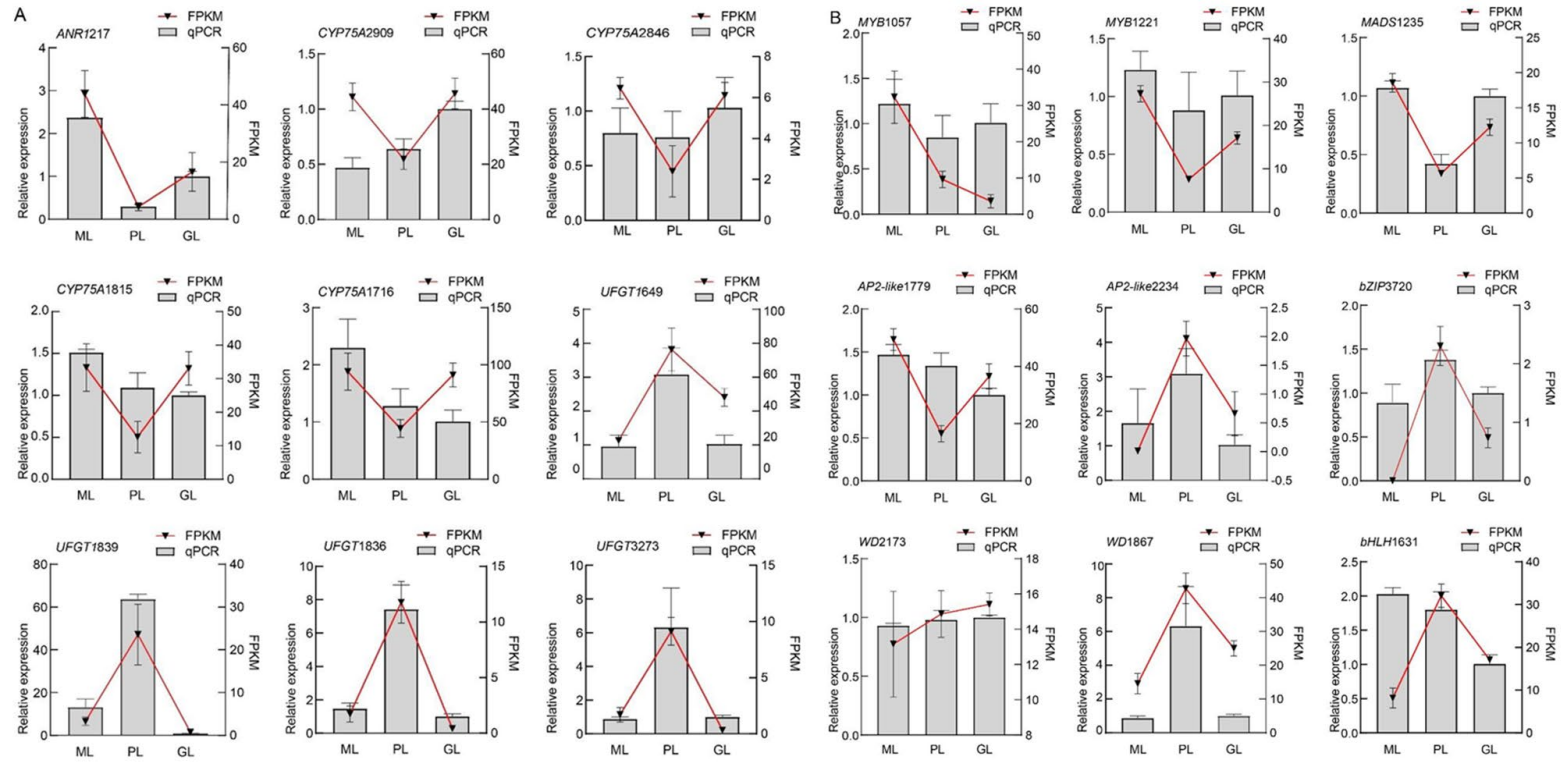
FPKM values calculated from the transcriptomic data, and transcriptional levels of flavonoid biosynthetic genes in the *L. chinense* var. *rubrum* detected by qRT-PCR analysis. The *β*-actin gene was used as an internal control. **A***ANR*, *UFGT*, and *CYP75A*. **B ***MYB*, *MADS-box*, *AP2-like*, *bZIP*, *WD*40 and *bHLH.* The control for relative expression GL was assigned the arbitrary value of 1.0. The data represent the six biological repeats and their average. Error bars represent the standard deviations of six biological replicates

To verify the expression levels of structural genes and transcription factors related to anthocyanin synthesis in *L. chinense* var. *rubrum*, we selected nine structural genes and nine transcription factors for qRT-PCR analysis (Additional file 12), and their correlation was evaluated (Additional file 13). *ANR* and *CYP75A* had higher expression levels in GL and ML than PL (Fig. 6A). Although *ANR* promotes the formation of (-)- epicatechin, it weakens the transformation ability of anthocyanins and leads to the formation of green leaves. ​In contrast, *UFGT* was highly expressed in PL leaves but had lower or no expression in ML and GL, respectively. Thus, we speculated that the upregulated expression of the *UFGT* gene might contribute to anthocyanin synthesis and regulate the formation of mosaic and purple leaves.

We verified expression trends of candidate transcription factors, such as *AP2-like2224*, *bZIP*3720, *WD*1867, *WD*2173, and *bHLH*1631; they were consistent with the *UFGT* gene expression trend with higher expression in the PL compared with GL and ML (Fig. 6B). At the same time, the Pearson correlation coefficient showed a strong correlation (R^2^ > 0.92) between the five transcription factors and the *UFGT* gene (Additional file 14), suggesting that these transcription factors were related to anthocyanin biosynthesis and regulated the appearance of leaf colour.

## Discussion

In ornamental plants with colourful leaves, research on leaf colour has always been the focus since it affects the ornamental quality and commercial value of the plants. However, the mechanism of leaf coloration in *L. chinense* var. *rubrum* was still unclear, necessitating its study using the existing technique.

**Fig. 7:**
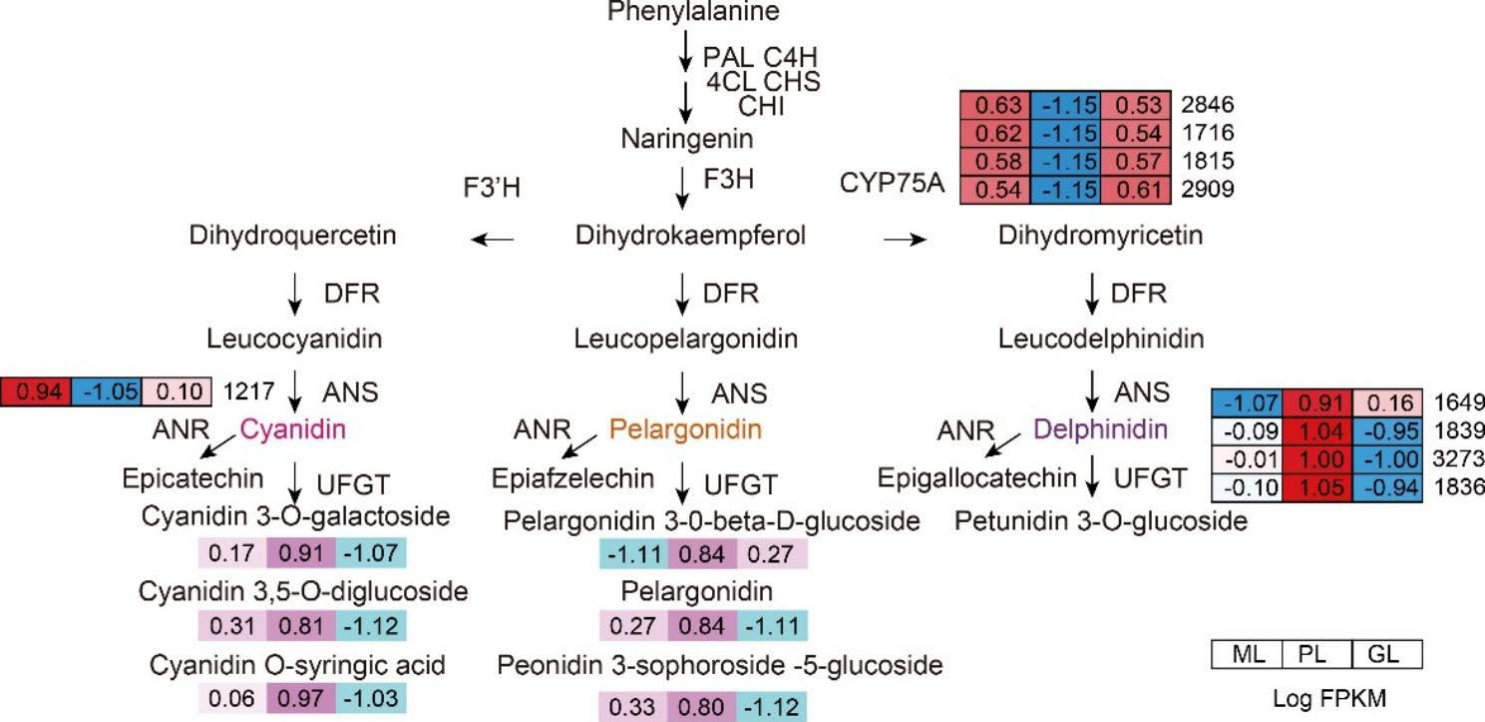
Putative genes in the anthocyanin synthesis pathway and their expression level. Heatmaps were constructed based on log2 (FPKM) of leaves ML, PL, and GL

In this study, anatomic and microscopic observations, pigment content determination, flavonoid metabolomics, and transcriptome sequencing were performed on *L. chinense* var. *rubrum* leaves of three different colours. Anatomic observations under the microscope showed that the mesophyll cells in ML were a mix of purple and green, those in PL were all purple, and those in GL were all green. On this basis, the determination of *L. chinense* var. *rubrum* pigment content proved the existence of chlorophyll, carotenoids, and anthocyanins in plant leaves. We speculated that ML had the pigments chlorophyll and anthocyanin, PL had anthocyanin and GL had chlorophyll. A total of 207 flavonoid compounds were detected using metabolomics. DEGs related to pigmentation were found in the transcriptome of the three-coloured leaves. To screen the main components and candidate genes of pigmentation, we proposed a hypothetical biosynthetic pathway (Fig. 7).

Our pigment content results showed that chlorophyll and carotenoid content was low relative to anthocyanins (Fig. 2). Therefore, differences in the type and content of anthocyanins were considered to be the possible reason for the different colours of *L. chinense* var. *rubrum* leaves.

At the metabolic level, anthocyanin biosynthesis may be the main pathway involved in leaf pigmentation (Additional file 4). We established that the total anthocyanin content of GL was lower than that of ML and PL and the change in the trend of anthocyanin content corresponded with the change in leaf colour. The contents of cyanidin 3-*O*-glucoside, cyanidin *O*-syringic acid, cyanidin 3,5-*O*-diglucoside, pelargonidin, petunidin 3-*O*-glucoside, and peonidin 3-sophoroside-5-glucoside were significantly different in the three leaves while those of peonidin, delphinidin, and cyanidin 3-*O*-rutinoside were not. Therefore, we speculate that the changes in the levels of these anthocyanins influence the colour of *L*. *chinense* var. *rubrum* leaves.

According to the differential expression of genes involved in anthocyanin synthesis and the difference in the content of various anthocyanins, we speculated the reasons for the different colour of *L*. *chinense* var. *rubrum* leaves. Although *CYP75A* was upregulated in ML and GL to accumulate the raw materials needed for anthocyanin synthesis, anthocyanin was converted into (-)-epicatechin due to the upregulation of *ANR*. Moreover, the downregulation of the *UFGT* gene ensured that anthocyanin could not be converted into a stable form, resulting in reduced anthocyanin content in the leaves. The *UFGT* gene in the anthocyanin biosynthesis pathway is the key to the formation of different types of anthocyanins [51]. In *Vitis vinifera*, the upregulation of *UFGT* genes led to the accumulation of anthocyanins [52]. Similar observations were made regarding *UFGT* expression and anthocyanin accumulation in *Malus pumila Mill* [53], *Litchi chinensis* [54], and mango [55]. Therefore, we speculate that *UFGT* genes contribute significantly to coloration, but the specific regulatory mechanism of these genes needs to be further verified.

In addition to structural genes, it is well known that transcription factors play an essential role in regulating the overall activity of flavonoid biosynthesis. *bHLH*, *MYB*, and *WD*40 proteins are the three main families responsible for regulating anthocyanin biosynthesis genes [56]. The results of qRT-PCR (Fig. 6B) and co-expression analysis (Additional file 14) demonstrated that the expressions of *AP2-Like*2234, *bZIP*3720, *bHLH*, and *WD*40 were upregulated in PL, correlating strongly with the late synergistic gene *UFGT*. We speculated that together; they played an important role in regulating the expression of anthocyanin synthesis genes in *L*. *chinense* var. *rubrum*.

At the same time, they may form ternary complexes of MBW to regulate the synthesis of anthocyanins [44, 57]. The MBW complex regulatory structural gene has been confirmed in *Petunia* and *Arabidopsis thaliana* and was shown to participate in the later steps of the anthocyanin and condensed tannin biosynthesis pathway [58–60]. *PpMYB* forms the MBW complex and especially activates *UFGT* to regulate the biosynthesis of anthocyanins [61, 62]. *Arabidopsis WD*-repeat/Mybs/*bHLH* complex, including *DFR*, *LDOX*, and *UF3GT*, mainly regulates the expression of ‘late’ anthocyanin biosynthesis genes [44]. Other transcription factors have also been implicated in this regulation: the *AP2-Like* gene in *Arabidopsis* [63] and *Solanum melongena* [64] the *MADS-box* protein in *Morellarubra* (red bayberry) and *Ficus carica L* [65] and the *bZIP* transcription factor in *Raphanus sativus L* (radish) [66]. These results indicated that these transcription factors might regulate the accumulation of anthocyanins by controlling the expression of differentially expressed *ANR*, *CYP75A*, and *UFGT* genes. In previous studies, *PpERF3* was shown to interact with *PpMYB114* and *PpbHLH3* to enhance the expression of *PpUFGT* [67]; *FaRAV1* directly bound to and activated *GT1* promoter to regulate anthocyanin accumulation [68]; *PybZIPa* promoted anthocyanin biosynthesis by regulating *PyMYB114*, *PyMYB10*, and *PyBBX22* as well as *PyUFGT* promoters [69]; *FaMADS1* played a negative role in the accumulation of anthocyanins in strawberry fruits by inhibiting structural genes [70]. It has been proved that transcription factors play a role in regulating the expression of structural genes, but the relationship with anthocyanin biosynthesis needs further experimental verification.

## Conclusion

In this study, the transcriptome and metabolome of three-coloured *L. chinense* var. *rubrum* leaves were sequenced and analyzed for the first time. We observed the content and dynamic changes in the levels of cyanidin 3-*O*-glucoside, cyanidin *O*-syringic acid, cyanidin 3,5-*O*-diglucoside, pelargonidin, petunidin 3-*O*-glucoside, and peonidin 3-sophoroside-5-glucoside influenced the colour variation in *L*. *chinense* var. *rubrum* leaves. Nine structural genes, such as *AN*R, *CYP75A*, and *UFGT*, and nine transcription factors, such as *MYB*, *MADS-box*, *AP2-Like*, *bZIP*, *WD*40, and *bHLH* were identified by KEGG database annotation and DEG analysis.

We combined the metabolome and transcriptome data to explore the molecular mechanism underlying *L*. *chinense* var. *rubrum* leaf coloration. By comparing ML, PL, and GL leaves, we speculate that significant upregulation of the *UFGT* gene is associated with the accumulation of anthocyanins. Therefore, these results may contribute to genetic modification or selection to further enhance the ornamental quality of *L*. *chinense* var. *rubrum*.

## Materials and methods

### Plant material

In this study, *L. chinense* var. *rubrum* plants were provided by Hunan Mid-subtropical Quality Plant Breeding and Utilization Engineering Technology Research Center, Hunan Agricultural University. We selected accession number Xiang S-SV-LC-032-2012-1 as the plant with mosaic leaves accession number Xiang S-SV-LC-032-2012-2 as the plant with purple leaves plant and accession number Xiang S-SV-LC-032-2012-3 as the plant with green leaves. We named them ML, PL, and GL, respectively. Plants were grown in the Garden Flower Base of Hunan Agricultural University, Changsha, Hunan Province, China. (28°12′N, 112°59′E). Samples were immediately frozen in liquid nitrogen after being collected and stored at -80℃ before metabolomic and transcriptomic analyses. The research materials were as shown in Fig. 2. Besides, apical middle and upper leaves per colour were selected; half the samples were sent to Wuhan Netware for metabolome determination and the metabolites were qualitatively and quantitatively analyzed based on the UPLC-Q-trap/MS detection platform and extensively targeted metabolome detection technology. The other half of the samples were sent to Beijing Novogene for transcriptome sequencing. Three biological replicates of GL, ML, and PL were recorded as samples GL1-3, ML1-3, and PL1-3.

## Analysis of leaf phenotype

The colour of the three-coloured leaves was measured with a Royal Horticultural Society Colour Chart and spectrophotometer, with seven leaves for each colour system. The brightness L*, the redness a*, and the yellowness b* were measured using the spectrophotometer, and the C* value representing the chroma was calculated by the formula: (CIRG) = (180 − *θ*)/(L*+C*), where C*=(a*^2^+b*^2^)^0.5^; hue angle *θ* represents the colour change, *θ* = arctan (*b* */*a* *) [71].

The whole leaf characteristics of the ML, PL, and GL were photographed under scattered light by a digital camera (Canon EOS5D Mark III, Japan). Then the lower epidermis of the leaves was torn off with forceps and the epidermal cells were observed under an eyepiece/objective lenses 10×/20× inverted microscope (Leica Microsystems CMS Gmbh, Germany). The mature leaves were sandwiched in two blades and cut; the cross-section was observed under an eyepiece/objective lenses 10×/20× inverted microscope (Leica Microsystems CMS Gmbh, Germany). The original resolution of all acquired microscopic images was 2048 × 1536.

Chlorophyll content and carotenoid content in GL, ML, and PL of *L. chinense* var. *rubrum* were measured directly by the extraction method. After the veins were removed, 0.1 g of each kind of leaf was weighed, followed by the addition of 15 ml of 95% ethanol was added. Every leaf tissue was incubated at 4℃ for extraction in the dark and finally, the supernatant was collected. The absorbance values at 470 nm, 649 nm, and 665 nm were measured with an ultraviolet spectrophotometer (AOE TSD-599, China) and each coloured-leaf variety had six replicates. Anthocyanin was detected by the pH differential method for cyanidin-3-glucoside content in plants. The revised method proposed by Zhang et al. was adopted [72] and the calculation formula is as follows: Ca = 13.95A_665_ − 6.88_649_; Cb = 24.96A_649_ − 7.32_A665_; Cc = (1000_A470_ − 2.05_Ca_ -114.8_Cb_)/248; TA = A*MW*5*100*V/e, where TA stands for total anthocyanin content (mg/100 g, as cyanidin-3-O-glucose equivalent), V stands for final volume (mL), and A = [A510 (pH 1.0) - A700 (pH 1.0)] - [A510 (pH 4.5) - A700 (pH 4.5)]. A molar absorptivity (e) of 26,900 m^2^.mol^− 1^ and molecular weight (MW) of 449.2 Da were used according to Wrolstad et al. (1982). Three measurements were taken for every six biological replicates [73].

## Metabolic analysis

Approximately 0.1 g of freeze-dried leaf sample approximately was weighed and pulverized with a grinder at 30 Hz for 1.5 min. Leaf metabolites were extracted by adding 1 ml extraction solution (70% of aqueous methanol) to an overnight incubation and 10 min centrifugation at 10,000 g at 4℃. The samples were filtered through a 0.22 μm membrane filter before analysis using an LC-ESI-MS/MS system (HPLC, Shim-pack UFLCSHIMADZU CBM30A system, www.shimadzu.com.cn/; MS, Applied Biosystems 4500 Q TRAP, www.appliedbiosystems.com.cn/). Chromatographic separation was performed on a Waters ACQUITY UPLC HSS T3 column (1.8 μm, 2.1 mm*100 mm) using solvent A (water, 0.04% acetic acid) and solvent B (acetonitrile, 0.04% acetic acid). The gradient program in terms of solvent system was solvent A: solvent B; the gradient program was 95:5 v/v at 0 min, 5:95 v/v at 11.0 min, 5:95 v/v at 12.0 min, 95:5 v/v at 12.1 min and 95:5 v/v at 15.0 min. The flow rate was 0.40 ml.min^− 1^, the column temperature was 40℃ and the injection volume was 5µL. The effluent was alternately connected to an electrospray-triple quadrupole rod-linear ion trap-mass spectrometer. The electrospray ionization source temperature was 550℃; the ion spray voltage was 5500 V and the CUR was set to 55, 60, and 25.0 psi respectively.

Based on the self-built database MWDB and the public database of metabolite information, the primary and secondary spectral data after mass spectrometric detection were qualitatively analyzed. The repetitive signals of high MW substances such as K^+^, Na^+^, and NH_3_^+^ were removed. The structure analysis of metabolites was from the Mass Bank (http://www.massbank.jp/), KNAPSACK (http://kanaya.naist.jp/KNAPSACK/), HMDB (http//www.hmdb.cal) [74], MOTODB (http://www.ab.wur.nl/moto) and METLIN (http://metlin.scripps.edu/index.php [75]. The KEGG pathway database (http://www.genome.jp/kegg/pathway.html) was used to identify related metabolic pathways. Metabolite quantification was performed using triple and four-stage mass spectrometric multiple reaction monitoring modes (MRM): the four-stage mass spectrometer first screened out the parent ion (Q1) of the target substance, which was further fragmented into fragment ions (Q2) and finally filtered through the three-stage four-stage mass spectrometer to screen out the characteristic fragment ions (Q3) [76].

## RNA extraction, library construction, and RNA-seq

Total RNA was extracted from approximately 2 g of *L. chinense* var. *rubrum* leaves using Gene Star (Beijing, China). Library preparation and transcriptome sequencing were performed by Novogene Bioinformatics Technology (Beijing, China). The libraries were sequenced using an Illumina HiSeq platform and the raw reads were filtered by the adaptor sequence, lower quality and N-containing reads followed by *de novo* assembly of the transcriptome using Trinity. The unigene for functional annotation was obtained by aligning the unigene with the GO, KOG, KEGG, Nt, Nr, and Pfam databases to annotate their potential metabolic pathways.

Expression levels of genes related to leaf colour formation in *L. chinense* var. *rubrum* were determined using the RSEM software package [77, 78]. The expression abundance of the unigenes was evaluated using the FPKM method [79]. Differential gene expression analyses in the different-coloured leaves were performed using the DESeq R package. DEGs were identified as those genes that had |log2(fold change)|≥2, FDR value < 0.01. GO and KEGG pathway enrichment analysis of the DEGs was done using the GOseq R package-based hypergeometric distribution [80], which can adjust for gene length bias in DEGs.

## qRT-PCR analysis

To further confirm the reliability of RNA-seq data in our differential expression analysis, the relative expression of nine structural genes in the flavonoid metabolic pathway was analyzed through qRT-PCR. Primers were designed using Primer Premier 5.0 software and the details are shown in Additional file 15. The *β*-actin gene was used as an internal reference gene and three biological replicates were set for each biological sample. The expression level of each gene in the list was calculated using the Livak method (delta-delta CT, 2^−ΔΔCt^)and expressed as the average standard deviation [81].

## Integrative metabolomic and transcriptomic analysis

Transcriptomic and metabolomic data for *L. chinense* var. *rubrum* leaves with clear differences were used for analysis. Correlation analysis was carried out according to the metabolite content of different colors of leaves in metabolic data and the differential gene expression in transcriptome data.

**Figure 1** Biosynthesis pathway of anthocyanin. PAL (phenylalanine ammonia lyase); C4H (cinnamate 4-monooxygenase); 4CL (4-coumarate-CoA ligase); CHS (chalcone synthase); CHI (chalcone isomerase); F3H (flavanone 3-hydroxylase); F3’H (flavonoid 3’-hydroxylase); F3’5’H (flavonoid 3’,5’-hydroxylase); DFR (dihydroflflavonol 4-reductase); ANS (anthocyanidin synthase); GT (glucosyltransferases); MT (methyltransferases) and AT (acyltransferases).

**Figure 4** Differential metabolites from different leaves. **A** PCA score plot of three materials and numbers of potential markers for each leaf color. **B** Venn diagram shows the overlapping and cultivar-specific differential metabolites from ML, PL, and GL.

**Figure 5** Differential expression genes in different colors. **A** Thermal diagram of the correlation coefficient between leaves. The Pearson correlation coefficient is within [-1, 1], and the closer it is to 1 or -1, the stronger the positive/anti-linear relationship. **B** Venn diagram of DEGs.

## Electronic supplementary material

Below is the link to the electronic supplementary material.

**Additional file 1: Table S1**. Detection Data of Various Physiological Characteristics of ML, PL, and GL Tricolor Leaves

**Additional file 2: Table S2.** A list of 207 flavonoid metabolites identified in *Loropetalum chinense* var. *rubrum*

**Additional file 3: Table S3.** Differential metabolites between GL, ML, and PL

**Additional file 4: Fig. S1.** KEGG annotation of putative proteins. The x-axis indicates the percentage of the number of genes annotated to the pathway out of the total number of genes annotated. The y-axis indicates the name of the KEGG metabolic pathway. **A** KEGG pathway analysis between GL and ML. **B** KEGG pathway analysis of between GL and PL. **C** KEGG pathway analysis between ML and PL

**Additional file 5: Table S4.** Statistical analysis of *L. chinense* var. *rubrum* reads in 9 libraries (Each sample was repeated three times)

**Additional file 6: Table S5.** The length distribution of assembled unigenes

**Additional file 7: Table S6.** The unigenes were successfully annotated to the seven databases

**Additional file 8: Table S7.** Number of DEGs in tricolor leaves

**Additional file 9: Fig. S2.** GO classification of DEGs. **A** GO functional classification of DEGs between GL vs. ML. **B** GO functional classification of DEGs between GL vs. PL. **C** GO functional classification of DEGs between ML vs. PL

**Additional file 10: Table S8.** KEGG annotation of different metabolic pathways

**Additional file 11: Table S9.** FPKM of key anthocyanin biosynthesis-related genes in leaves of *L. chinense* var. *rubrum*

**Additional file 12: Table S10.** qRT-PCR data for structural and transcriptional factors

**Additional file 13: Table S11.** R2 of key anthocyanin biosynthesis-related

**Additional file 14: Fig. S3.** Correlation analysis of transcription factors and structural genes

**Additional file 15: Table S12.** List of primers used in this study

## Data Availability

All data generated/analyzed during this study are included in this article and its supplementary files. The sequencing data associated with transcription profiles in this study have been deposited in the NCBI SRA database with accession number PRJNA741349(https://www.ncbi.nlm.nih.gov/sra/ PRJNA741349). The metabolomics data have been uploading in Metabolites database with accession number MTBLS5662 (https://www.ebi.ac.uk/metabolights/MTBLS5662/descriptors).

## Abbreviations

*L. chinense* var. *rubrum*: *Loropetalum chinense* var. *rubrum*
GL: green leaves
ML: mosaiced leaves
PL: purple leaves
Ca: Chlorophyll a
Cb: Chlorophyll b
Cc: Carotenoid concentration
TA: Total anthocyanin
FW: fresh weight
*PAL*: phenylalanine ammonia-lyase
*C4H*: cinnamate 4-hydroxylase
*4CL*: 4-coumaroyl:CoA-ligase
*CHS*: chalcone synthase
CHI: chalcone isomerase
*F3H*: flavanone 3-hydroxylase
*ANR*: anthocyanidin reductase
*CYP75A*: flavonoid 3’,5’-hydroxylase
*DFR*: dihydro-flavonol 4-reductase
UDP-glucose: flavonoid-3-*O-*glycosyltransferase
*MYB*: *MYB* transcription factor
*bHLH*: basic helix-loop-helix transcription factor
*WD*40: *WD*40 proteins
*bZIP*: *bZIP* transcription factor
*AP2-like*: *AP2/ERF*
MASD: *MADS-box* transcription factor
Log2FC: Log2 fold change
DEGs: differentially expressed genes
GO: Gene ontology
KOG: eukaryotic Ortholog Groups
KEGG: Kyoto Encyclopedia of Gene and Genomes
Nt: NCBI nucleotide sequences
Nr: NCBI nonredundant protein
Pfam: Protein family
MW: Molecular weight
MRM: Multiple reaction monitoring
CUR: Curtain gas
MWDB: Met ware database
