# Supplementary material for: Comprehensive analysis of metabolome and transcriptome reveals the mechanism of color formation in different leave of Loropetalum Chinense var. Rubrum
Source: BMC Plant Biol. 2023 Mar 8;23:133. doi: 10.1186/s12870-023-04143-9 (PMC9993627; doi:10.1186/s12870-023-04143-9)
Supplement: Supplementary file 1 — Additional file 1: Table S1. Detection Data of Various Physiological Characteristics of ML, PL, and GL Tricolor Leaves [file 12870_2023_4143_MOESM1_ESM.docx]

**Additional file 4: Fig. S1.**

**
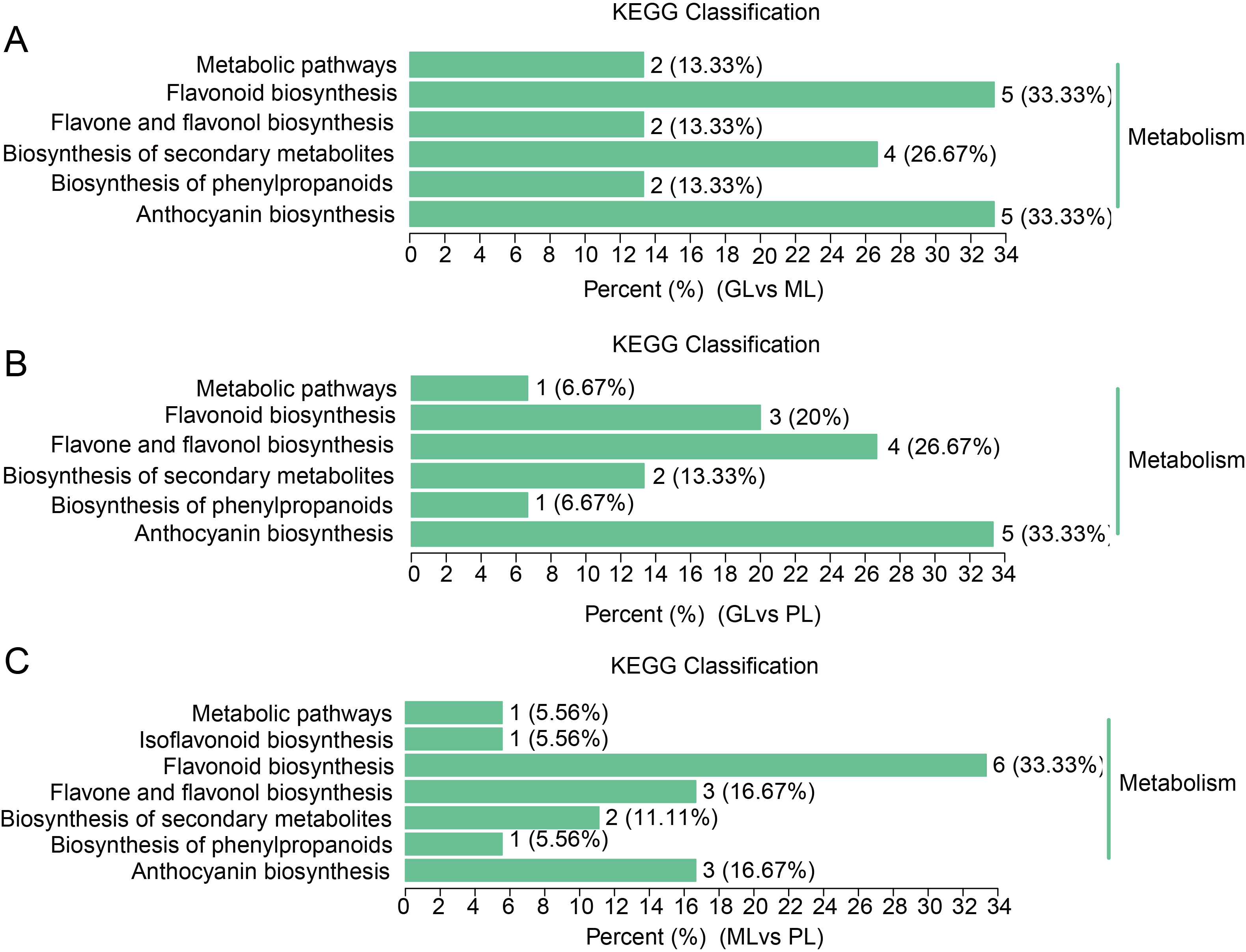
**

**Fig. S1.** KEGG annotation of putative proteins. The x-axis indicates the percentage of the number of genes annotated to the pathway out of the total number of genes annotated. The y-axis indicates the name of KEGG metabolic pathway. **A** KEGG pathway analysis of between GL and ML**. B** KEGG pathway analysis of between GL and PL. **C** KEGG pathway analysis of between ML and PL.
