# Supplementary material for: Comprehensive analysis of metabolome and transcriptome reveals the mechanism of color formation in different leave of Loropetalum Chinense var. Rubrum
Source: BMC Plant Biol. 2023 Mar 8;23:133. doi: 10.1186/s12870-023-04143-9 (PMC9993627; doi:10.1186/s12870-023-04143-9)
Supplement: Supplementary file 2 — Additional file 2: Table S2. A list of 207 flavonoid metabolites identified in Loropetalum chinense var. rubrum [file 12870_2023_4143_MOESM2_ESM.docx]

**Additional files 6:Table S5.**

Table S5.The length distribution of assembled unigenes

| **Length range (bp)** | **Unigenes** |
| --- | --- |
| <500 | 110,962 |
| 500-1000 | 31,726 |
| 1000-2000 | 38,050 |
| 2000-3000 | 23,499 |
| >3000 | 27,573 |
| Total number | 231,810 |
| Total nucleotides | 294,494,833 |
| Max length | 21,135 |
| Mean length | 1271 |
| N50 | 2608 |
