# Supplementary material for: Comprehensive analysis of metabolome and transcriptome reveals the mechanism of color formation in different leave of Loropetalum Chinense var. Rubrum
Source: BMC Plant Biol. 2023 Mar 8;23:133. doi: 10.1186/s12870-023-04143-9 (PMC9993627; doi:10.1186/s12870-023-04143-9)
Supplement: Supplementary file 3 — Additional file 3: Table S3. Differential metabolites between GL, ML, and PL [file 12870_2023_4143_MOESM3_ESM.docx]

**Additional files 7:Table S6.**

Table S6.The unigenes were successfully annotated to the seven databases.

| **Database** | **Annotation num** |
| --- | --- |
| NR | 118,518 |
| SwissProt | 96,572 |
| KEGG | 115,058 |
| KOG | 75,254 |
| GO | 84,662 |
| NT | 88,001 |
| Pfam | 84,662 |
