# Supplementary material for: Comprehensive analysis of metabolome and transcriptome reveals the mechanism of color formation in different leave of Loropetalum Chinense var. Rubrum
Source: BMC Plant Biol. 2023 Mar 8;23:133. doi: 10.1186/s12870-023-04143-9 (PMC9993627; doi:10.1186/s12870-023-04143-9)
Supplement: Supplementary file 4 — Additional file 4: Fig. S1. KEGG annotation of putative proteins. The x-axis indicates the percentage of the number of genes annotated to the pathway out of the total number of genes annotated. The y-axis indicates the name of the KEGG metabolic pathway. A KEGG pathway analysis between GL and ML. B KEGG pathway analysis of between GL and PL. C KEGG pathway analysis between ML and PL [file 12870_2023_4143_MOESM4_ESM.docx]

**Additional files 8:Table S7**

| Groups | Total | Down | Up |
| --- | --- | --- | --- |
| GL vs ML | 5646 | 3447 | 2199 |
| GL vs PL | 1217 | 539 | 678 |
| ML vs PL | 6838 | 2613 | 4223 |

Table S7. Number of DEGs in tricolor leaves.
