# Supplementary material for: Comprehensive analysis of metabolome and transcriptome reveals the mechanism of color formation in different leave of Loropetalum Chinense var. Rubrum
Source: BMC Plant Biol. 2023 Mar 8;23:133. doi: 10.1186/s12870-023-04143-9 (PMC9993627; doi:10.1186/s12870-023-04143-9)
Supplement: Supplementary file 6 — Additional file 6: Table S5. The length distribution of assembled unigenes [file 12870_2023_4143_MOESM6_ESM.docx]

| **Gene names** | **FPKM**  **GL** | **FPKM**  **PL** | **FPKM**  **ML** |
| --- | --- | --- | --- |
| *ANR*1217 | 16.56±2.24 b | 4.37±0.45 c | 43.85±2.73 a |
| *CYP75A*2846 | 6.11±0.37 a | 2.40±0.72 b | 6.46±0.30 a |
| *CYP75A*1716 | 91.23±6.02 a | 44.33±4.4 b | 94.05±9.34 a |
| *CYP75A*1815 | 33.06±2.90 a | 12.59±2.71 b | 33.31±4.09 a |
| *CYP75A*2909 | 45.63±3.19 a | 21.95±2.14 b | 44.41±2.88 a |
| *UFGT*1649 | 48.10±3.02 b | 76.29±7.27 a | 22.67±0.52 c |
| *UFGT*1839 | 0.75±0.18 b | 23.58±4.11 a | 3.28±0.56 b |
| *UFGT*3273 | 0.33±0.12 b | 9.13±0.72 a | 1.70±0.38 b |
| *UFGT*1836 | 0.42±0.13 b | 11.77±1.08 a | 1.73±0.41 b |
| *MYB*1057 | 3.61±1.06 b | 9.62±1.31 b | 32.33±4.17 a |
| *MYB*1221 | 17.1±0.82 b | 7.56±0.44 c | 27.31±1.09 a |
| *MADS*1235 | 12.23±0.67 b | 5.63±0.24 c | 18.55±0.76 a |
| *AP2like*1799 | 36.49±2.51b | 16.53±1.64 c | 49.37±2.20 a |
| *AP2like*2234 | 0.66±0.22 b | 1.96±0.17 a | 0.01±0.01 c |
| *bZIP*3720 | 0.74±0.10 b | 2.30±0.19 a | 0±0 c |
| *WD*2173 | 15.41±0.37 | 14.87±0.77 | 13.15±1.73 |
| *WD*1867 | 25.04±1.29 b | 42.75±2.61 a | 14.55±1.76 c |
| *bHLH*1631 | 17.07±0.42 b | 32.09±1.58 a | 8.15±1.35 c |

**Additional files 11:Table S9.**

Table S9. FPKM of key anthocyanin biosynthesis related genes in leaves of *L. chinense* var*. rubrum*

Note: Each sample was detected with three biological repetitions. Mean (±SE) with different lower letters are significantly different within the (mean separation by LSD and Duncan’s test at P < 0.05).
