# Supplementary material for: Comprehensive analysis of metabolome and transcriptome reveals the mechanism of color formation in different leave of Loropetalum Chinense var. Rubrum
Source: BMC Plant Biol. 2023 Mar 8;23:133. doi: 10.1186/s12870-023-04143-9 (PMC9993627; doi:10.1186/s12870-023-04143-9)
Supplement: Supplementary file 9 — Additional file 9: Fig. S2. GO classification of DEGs. A GO functional classification of DEGs between GL vs. ML. B GO functional classification of DEGs between GL vs. PL. C GO functional classification of DEGs between ML vs. PL [file 12870_2023_4143_MOESM9_ESM.docx]

**Additional files 15:Table S12.**

Table S12.List of primers used in this study

| **Gene names** | **primer sequences(5'-3')** |
| --- | --- |
| ***ANR*1217** | F:CAGATAGACATAGAAGCAGAAC |
|  | R:TTCACATAATGAGGTCCATAATC |
| ***CYP75A*2909** | F:CTGGAGGAGTCTGACATAC |
|  | R:GGAAGATTGAGTGGTGTTG |
| ***CYP75A*2846** | F:GATGGATAAGGTGATTGGAAG |
|  | R:GGAAGATTGAGTGGTGTTG |
| ***CYP75A*1716** | F:GATGGATAAGGTGATTGGAAG |
|  | R:GGAAGATTGAGTGGTGTTG |
| ***CYP75A*1815** | F:CTGGAGGAGTCTGACATAC |
|  | R:GATTGAGTGGTGTTGAAGG |
| ***UFGT*1649** | F:GTGGCTTCTGCTTCTCTA |
|  | R:AACATCAACAGTCCATCCTA |
| ***UFGT*1839** | F:TTGATAGCAACCGACCTT |
|  | R:TCCTTCTTGTTCCAATTCTTC |
| ***UFGT*1836** | F:AATCCGTTAGACCATACATTATAC |
|  | R:TCACCAAAAAATGGCCGGCAAATTA |
| ***UFGT*3273** | F:CCCAACAATAACAAATGACTTC |
|  | R:CCATAAGCATCGGAACTTG |
| ***MYB1057*** | F:GTCAGATTATGAATACCTCACAA |
|  | R:ATGCCATTCCTTCAATATAGC |
| ***MYB1221*** | F:AAGAACTATTGGAACACTCATC |
|  | R:CTACACTTGGCTCACTACT |
| ***MADS-BOX1235*** | F:AAGAAGAAGAACAAGAAGAAGAA |
|  | R:ATGAGCATCACAGAGAACT |
| ***AP2-Like1779*** | F:AGTGGAGTTGAGTTAGGTAAT |
|  | R:TCTTGTGTTATGTGCTGTTC |
| ***AP2-Like2234*** | F:AACAACAACAATGGAGTAATAGA |
|  | R:ATCATCATCATCATCGTAATGG |
| ***bZIP3720*** | F:GTGGATGCTTGAATGATAGG |
|  | R:GTGTGTAGAATGGTTTGAGAA |
| ***WD2173*** | F:GTCATTCATTGGTCTTCATTAAC |
|  | R:GCAACAAGCAACCTATCTT |
| ***WD1867*** | F:GAGGATACTGTTGAAGATGTG |
|  | R:CCATAATATAAGGCGAGAATCAT |
| ***BHLH1631*** | F:TGACTTCGGTTGATGAGAT |
|  | R:ATGATATACGCAGGTAAGGAT |
| ***Actin2*** | F:CCACCAGGCTTATTGATAGAAT |
|  | R:CAATGGTTGAACCTGAATACT |
