# Supplementary material for: Comprehensive analysis of metabolome and transcriptome reveals the mechanism of color formation in different leave of Loropetalum Chinense var. Rubrum
Source: BMC Plant Biol. 2023 Mar 8;23:133. doi: 10.1186/s12870-023-04143-9 (PMC9993627; doi:10.1186/s12870-023-04143-9)
Supplement: Supplementary file 10 — Additional file 10: Table S8. KEGG annotation of different metabolic pathways [file 12870_2023_4143_MOESM10_ESM.docx]

**Additional files 14: Fig. S3.**


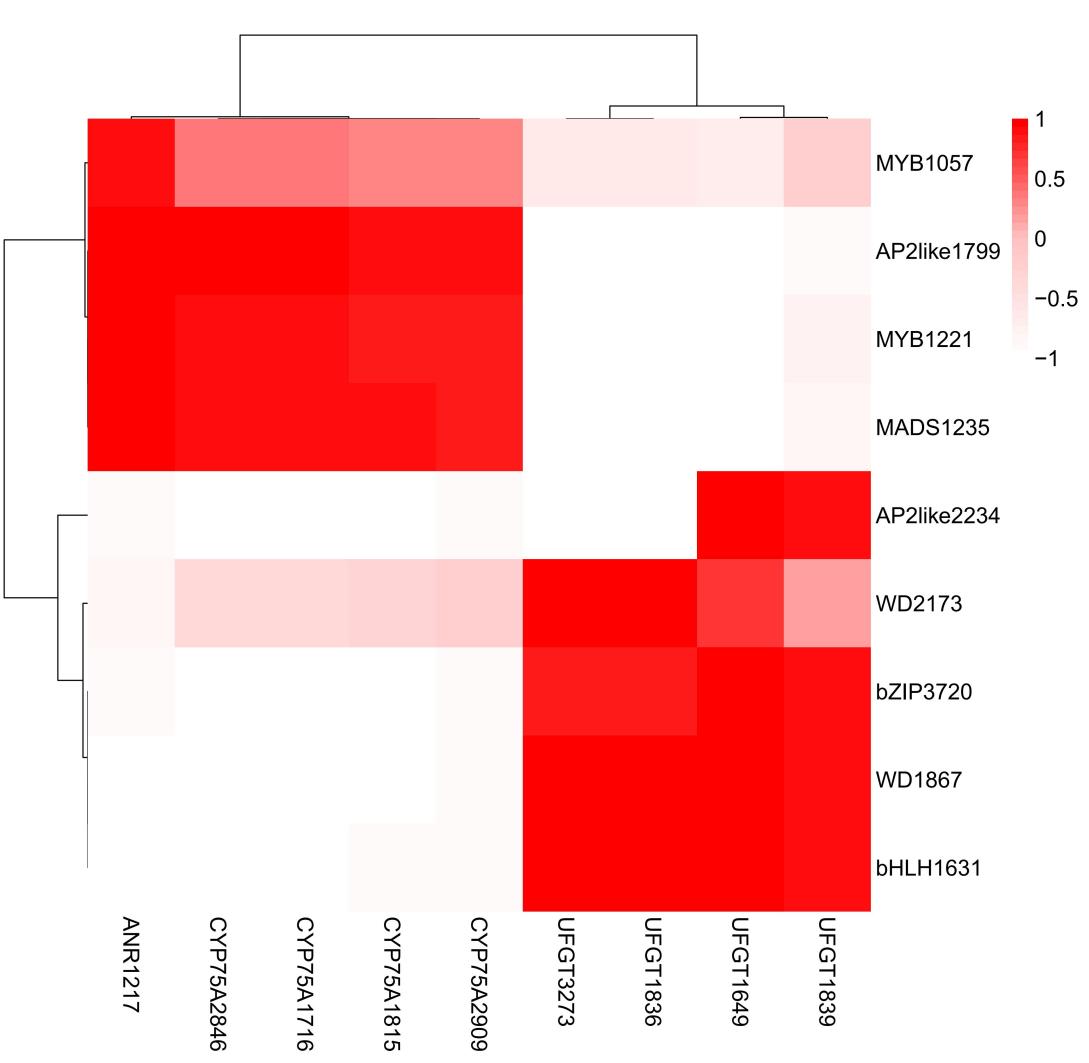


**Fig. S3.** Correlation analysis of transcription factors and structural genes
