# Supplementary material for: Comprehensive analysis of metabolome and transcriptome reveals the mechanism of color formation in different leave of Loropetalum Chinense var. Rubrum
Source: BMC Plant Biol. 2023 Mar 8;23:133. doi: 10.1186/s12870-023-04143-9 (PMC9993627; doi:10.1186/s12870-023-04143-9)
Supplement: Supplementary file 12 — Additional file 12: Table S10. qRT-PCR data for structural and transcriptional factors [file 12870_2023_4143_MOESM12_ESM.docx]

**Additional files 2:Table S2.**

Table S2.A list of 207 flavonoid metabolites identified in *Loropetalum chinense* var. *rubrum*.

| **Index** | **Q1 (Da)** | **Q3 (Da)** | **Rt (min)** | **Molecular Weight (Da)** | **Ionization model** | **KEGG ID** | **Compounds** | **Class** |
| --- | --- | --- | --- | --- | --- | --- | --- | --- |
| pma0249 | 479.118 | 317.1 | 3.6 | 478.118 | [M+H]+ | - | Selgin 5-O-hexoside | Anthocyanins |
| pma0253 | 477.14 | 315 | 5.17 | 476.14 | [M+H]+ | - | O-methylChrysoeriol 5-O-hexoside | Anthocyanins |
| pma0724 | 435.1 | 285.1 | 3.73 | 434.1 | [M+H]+ | - | Naringenin C-hexoside | Anthocyanins |
| pma0760 | 565 | 317 | 4.28 | 564 | [M+H]+ | - | Selgin O-malonylhexoside | Anthocyanins |
| pma0779 | 725.2 | 331.1 | 4.48 | 724.2 | [M+H]+ | - | Tricin O-rhamnosyl-O-malonylhexoside | Anthocyanins |
| pma0787 | 551.1 | 181.1 | 4.4 | 550.1 | [M+H]+ | - | Quercetin-3-(6''-malonyl)-Glucoside | Anthocyanins |
| pma1108 | 433.113 | 283.1 | 3.73 | 432.113 | [M+H]+ | - | Apigenin C-glucoside | Anthocyanins |
| pma1116 | 301.1 | 286 | 4.06 | 300.1 | [M+H]+ | C10098 | Kaempferide | Anthocyanins |
| pma6218 | 419.1 | 383.1 | 3.7 | 418.1 | [M+H]+ | - | O-methylnaringenin C-pentoside | Anthocyanins |
| pma6389 | 345.2 | 177.2 | 6.27 | 344.2 | [M+H]+ | C04444 | Ayanin | Anthocyanins |
| pma6496 | 449.1 | 299.1 | 3.41 | 448.1 | [M+H]+ | - | Luteolin 6-C-glucoside | Anthocyanins |
| pma6515 | 755.2 | 177.1 | 4.6 | 754.2 | [M+H]+ | - | C-hexosyl-chrysin O-feruloylhexoside | Anthocyanins |
| pma6558 | 313.08 | 298.05 | 7.31 | 314.08 | [M-H]- | - | Velutin | Anthocyanins |
| pma6576 | 347 | 153.2 | 4.3 | 346 | [M+H]+ | - | Spinacetin | Anthocyanins |
| pma6638 | 477.2 | 315 | 5.4 | 476.2 | [M+H]+ | - | O-methylChrysoeriol 7-O-hexoside | Anthocyanins |
| pma6639 | 479.2 | 317.2 | 4.11 | 478.2 | [M+H]+ | - | Isorhamnetin O-hexoside | Flavanone |
| pma6647 | 625.2 | 607.1 | 3.4 | 624.2 | [M+H]+ | - | C-hexosyl-chrysoeriol O-hexoside | Flavanone |
| pmb0550 | 449.1 | 287.3 | 2.55 | 449.1 | Protonated | C08604 | Cyanidin 3-O-glucoside (Kuromanin) | Flavanone |
| pmb0563 | 301.1 | 286 | 3.94 | 301.1 | Protonated | C08726 | Peonidin | Flavanone |
| pmb0565 | 509.1 | 347.2 | 3.36 | 508.1 | [M+H]+ | - | Syringetin 3-O-hexoside | Flavanone |
| pmb0566 | 581.2 | 383.2 | 3.7 | 580.2 | [M+H]+ | - | Luteolin O-hexosyl-O-pentoside | Flavanone |
| pmb0576 | 519 | 271 | 4.36 | 518 | [M+H]+ | - | Apigenin O-malonylhexoside | Flavanone |
| pmb0579 | 669 | 207 | 4.88 | 668 | [M+H]+ | - | Chrysoeriol O-sinapoylhexoside | Flavanone |
| pmb0580 | 417.1 | 255.1 | 4.94 | 416.1 | [M+H]+ | - | Chrysin 5-O-glucoside (Toringin) | Flavanone |
| pmb0588 | 611.1 | 449.3 | 3.44 | 610.1 | [M+H]+ | - | "Luteolin 3',7-di-O-glucoside" | Flavanone |
| pmb0592 | 771.1 | 463.2 | 3.78 | 770.1 | [M+H]+ | - | Chrysoeriol O-hexosyl-O-rutinoside | Flavanone |
| pmb0595 | 479.2 | 317.2 | 4.09 | 478.2 | [M+H]+ | - | Isorhamnetin 5-O-hexoside | Flavanone |
| pmb0600 | 609.4 | 301 | 4 | 608.4 | [M+H]+ | - | Chrysoeriol 7-O-rutinoside | Flavanone |
| pmb0602 | 509.3 | 347.2 | 4.08 | 508.3 | [M+H]+ | - | Syringetin 7-O-hexoside | Flavanone |
| pmb0604 | 449.1 | 287.6 | 4.04 | 448.1 | [M+H]+ | C12249 | Kaempferol 3-O-glucoside (Astragalin) | Flavanone |
| pmb0618 | 627.1 | 465.1 | 2.7 | 626.1 | [M+H]+ | - | 8-C-hexosyl-hesperetin O-hexoside | Flavanone |
| pmb0622 | 611.2 | 473.1 | 2.86 | 610.2 | [M+H]+ | - | C-hexosyl-luteolin O-hexoside | Flavanone |
| pmb0624 | 611.1 | 431.3 | 2.99 | 610.1 | [M+H]+ | - | 6-C-hexosyl-luteolin O-hexoside | Flavanone |
| pmb0628 | 613.1 | 451.1 | 3 | 612.1 | [M+H]+ | - | Eriodictiol C-hexosyl-O-hexoside | Flavanone |
| pmb0629 | 463.1 | 313.1 | 3.03 | 462.1 | [M+H]+ | - | Chrysoeriol 6-C-hexoside | Flavanone |
| pmb0645 | 627.1 | 465.2 | 3.38 | 626.1 | [M+H]+ | - | 6-C-hexosyl-hesperetin O-hexoside | Flavone |
| pmb0647 | 581.1 | 419.1 | 3.45 | 580.1 | [M+H]+ | - | 8-C-hexosyl-luteolin O-pentoside | Flavone |
| pmb0653 | 595.1 | 463.1 | 3.61 | 594.1 | [M+H]+ | - | "di-C,C-hexosyl-apigenin" | Flavone |
| pmb0660 | 757.2 | 147.1 | 3.66 | 756.2 | [M+H]+ | - | C-hexosyl-luteolin O-p-coumaroylhexoside | Flavone |
| pmb0663 | 611.1 | 317.1 | 3.68 | 610.1 | [M+H]+ | - | 8-C-hexosyl-luteolin O-hexoside | Flavone |
| pmb0665 | 611.2 | 465.1 | 3.77 | 610.2 | [M+H]+ | - | Luteolin 8-C-hexosyl-O-hexoside | Flavone |
| pmb0689 | 463.1 | 331.2 | 4.66 | 462.1 | [M+H]+ | - | Chrysoeriol C-hexoside | Flavone |
| pmb0696 | 625 | 463 | 3.4 | 624 | [M+H]+ | - | 8-C-hexosyl chrysoeriol O-hexoside | Flavone |
| pmb0701 | 463.1 | 313.2 | 3.81 | 462.1 | [M+H]+ | - | Chrysoeriol 8-C-hexoside | Flavone |
| pmb0711 | 611.2 | 303.1 | 3.63 | 610.2 | [M+H]+ | - | Quercetin 7-O-rutinoside | Flavone |
| pmb0713 | 655.2 | 331.2 | 3.42 | 654.2 | [M+H]+ | - | Tricin 7-O-hexosyl-O-hexoside | Flavone |
| pmb0724 | 477.1 | 331.1 | 4.9 | 476.1 | [M+H]+ | - | Tricin O-rhamnoside | Flavone |
| pmb0725 | 669.1 | 331.4 | 5 | 668.1 | [M+H]+ | - | Tricin 7-O-feruloylhexoside | Flavone |
| pmb0732 | 669.1 | 331 | 3.77 | 668.1 | [M+H]+ | - | Tricin 5-O-feruloylhexoside | Flavone |
| pmb0739 | 659.3 | 331 | 4.83 | 658.3 | [M+H]+ | - | Tricin O-hexosyl-O-syringin alcohol | Flavone |
| pmb0746 | 527.1 | 331 | 5.75 | 526.1 | [M+H]+ | - | Tricin 4'-O-β-guaiacylglycerol | Flavone |
| pmb0835 | 611.2 | 287.1 | 2.43 | 610.2 | [M+H]+ | - | Gallocatechin-gallocatechin | Flavone |
| pmb2831 | 315.1 | 153.1 | 2.52 | 316.1 | [M-H]- | - | Protocatechuic acid O-glucoside | Flavone |
| pmb2850 | 329 | 314 | 5.66 | 330 | [M-H]- | - | Tricin | Flavone |
| pmb2947 | 865.1 | 407.1 | 3.54 | 866.1 | [M-H]- | - | Catechin-catechin-catechin | Flavone |
| pmb2957 | 465.1 | 285.1 | 2.54 | 466.1 | [M-H]- | - | Cyanidin O-syringic acid | Flavone |
| pmb2975 | 477.1 | 357.1 | 3.45 | 478.1 | [M-H]- | - | Hesperetin O-Glucuronic acid | Flavone |
| pmb2979 | 549.2 | 387.1 | 3.96 | 550.2 | [M-H]- | - | Hesperetin O-malonylhexoside | Flavone |
| pmb2983 | 723.1 | 311.6 | 4.61 | 724.1 | [M-H]- | - | Chrysoeriol C-pentosyl-O-rhamnosyl-rhamnoside | Flavone |
| pmb2987 | 487.1 | 163 | 3.08 | 488.1 | [M-H]- | - | Acacetin O-acetyl hexoside | Flavone |
| pmb2992 | 459.1 | 255.2 | 3.71 | 460.1 | [M-H]- | - | Acacetin O-glucuronic acid | Flavone |
| pmb2999 | 461.1 | 299.1 | 3.97 | 462.1 | [M-H]- | - | Chrysoeriol 5-O-hexoside | Flavone |
| pmb3006 | 431.1 | 269.1 | 4.13 | 432.1 | [M-H]- | C04608 | Apigenin 7-O-glucoside (Cosmosiin) | Flavone |
| pmb3013 | 519.1 | 314.2 | 4.26 | 520.1 | [M-H]- | - | Isorhamnetin O-acetyl-hexoside | Flavone |
| pmb3014 | 641.1 | 285.3 | 4.57 | 642.1 | [M-H]- | - | Luteolin O-eudesmic acid-O-hexoside | Flavone |
| pmb3023 | 449.1 | 287 | 3.29 | 450.1 | [M-H]- | - | Eriodictyol C-hexoside | Flavone |
| pmb3024 | 447.1 | 327.1 | 3.38 | 448.1 | [M-H]- | - | Luteolin C-hexoside | Flavone |
| pmb3026 | 505.1 | 301.2 | 3.9 | 506.1 | [M-H]- | - | Quercetin O-acetylhexoside | Flavone |
| pmb3037 | 571.1 | 329.1 | 5.73 | 572.1 | [M-H]- | - | Tricin O-malonyl shikimic acid | Flavone |
| pmb3041 | 521.1 | 329.2 | 3.75 | 522.1 | [M-H]- | - | Tricin O-saccharic acid | Flavone |
| pmb3045 | 505.1 | 329.1 | 4.11 | 506.1 | [M-H]- | - | Tricin O-glucuronic acid | Flavone |
| pmb3046 | 491.1 | 315.1 | 4.18 | 492.1 | [M-H]- | - | Tricin 7-O-hexoside | Flavone |
| pmb3047 | 657.1 | 495.1 | 4.5 | 658.1 | [M-H]- | - | Tricin 4'-O-(syringyl alcohol) ether 5-O-hexoside | Flavone |
| pmb3114 | 561.1 | 289.2 | 3.63 | 562.1 | [M-H]- | - | Epicatechin-epiafzelechin | Flavone |
| pmb3894 | 329.1 | 229.1 | 5.82 | 330.1 | [M-H]- | - | Di-O-methylquercetin | Flavone |
| pmc1990 | 299.1 | 223.1 | 6.72 | 300.1 | [M-H]- | - | "4'-Hydroxy-5,7-dimethoxyflavanone" | Flavone |
| pme0001 | 609.2 | 301 | 4.22 | 610.19 | [M-H]- | C09806 | Hesperetin 7-O-neohesperidoside (Neohesperidin) | Flavone |
| pme0088 | 285 | 151 | 5.02 | 286 | [M-H]- | C01514 | Luteolin | Flavone |
| pme0197 | 609.153 | 301 | 3.61 | 610.153 | [M-H]- | C05625 | Quercetin 3-O-rutinoside (Rutin) | Flavone |
| pme0200 | 287.048 | 153 | 5.66 | 286.048 | [M+H]+ | C05903 | Kaempferol | Flavone |
| pme0205 | 289.079 | 245 | 2.96 | 290.079 | [M-H]- | C06562 | Catechin | Flavone |
| pme0208 | 537.09 | 375 | 5.91 | 538.09 | [M-H]- | C10018 | Amentoflavone | Flavone |
| pme0324 | 255.058 | 153 | 6.87 | 254.0579 | [M+H]+ | C10028 | Chrysin | Flavone |
| pme0333 | 579.164 | 271 | 4 | 578.1636 | [M+H]+ | C12627 | Apigenin 7-O-neohesperidoside (Rhoifolin) | Flavone |
| pme0355 | 255.058 | 137 | 4.85 | 254.0579 | [M+H]+ | C10208 | Daidzein | Flavone |
| pme0359 | 433.106 | 271.01 | 3.93 | 432.1056 | [M+H]+ | - | Apigenin 5-O-glucoside | Flavone |
| pme0361 | 435.085 | 303 | 4.03 | 434.0849 | [M+H]+ | - | Quercetin 3-alpha-L-arabinofuranoside (Avicularin) | Flavone |
| pme0363 | 299.063 | 284.01 | 5.7 | 300.0634 | [M-H]- | C04293 | Chrysoeriol | Flavone |
| pme0367 | 577.164 | 269.01 | 3.99 | 578.1636 | [M-H]- | - | Apigenin 7-rutinoside (Isorhoifolin) | Flavone |
| pme0369 | 593.159 | 285 | 3.86 | 594.1585 | [M-H]- | - | Kaempferol 3-O-rutinoside (Nicotiflorin) | Flavone |
| pme0372 | 435.121 | 273 | 4.18 | 434.1213 | [M+H]+ | C09099 | Naringenin 7-O-glucoside (Prunin) | Flavone |
| pme0376 | 271.069 | 151 | 5.56 | 272.0685 | [M-H]- | C00509 | Naringenin | Flavone |
| pme0379 | 271.053 | 153.01 | 5.57 | 270.0528 | [M+H]+ | C01477 | Apigenin | Flavone |
| pme0431 | 577 | 287 | 3.62 | 576.1268 | [M+H]+ | - | Procyanidin A1 | Flavone |
| pme0434 | 577 | 407.1 | 3.04 | 578.1424 | [M-H]- | - | Procyanidin B2 | Flavone |
| pme0436 | 577.1 | 407.1 | 2.83 | 578.1424 | [M-H]- | - | Procyanidin B3 | Flavone |
| pme0442 | 303 | 229 | 2.96 | 303.24 | Protonated | C05908 | Delphinidin | Flavone |
| pme0450 | 289 | 245 | 3.26 | 290.3 | [M-H]- | C09727 | L-Epicatechin | Flavone |
| pme1201 | 273.084 | 167 | 5.49 | 274.084 | [M-H]- | C00774 | Phloretin | Flavone |
| pme1398 | 465.1 | 303 | 2.41 | 465.1 | Protonated | C12138 | Delphinidin 3-O-glucoside (Mirtillin) | Flavone |
| pme1478 | 317 | 151 | 4.42 | 318.038 | [M-H]- | C10107 | Myricetin | Flavone |
| pme1486 | 457.2 | 169 | 3.28 | 458.085 | [M-H]- | C09731 | Epigallate catechin gallate (EGCG) | Flavone |
| pme1500 | 313.079 | 283 | 7.32 | 314.079 | [M-H]- | - | Kumatakenin | Flavone |
| pme1506 | 433.106 | 287 | 4.87 | 432.106 | [M+H]+ | - | Kaempferol 7-O-rhamnoside | Flavone |
| pme1510 | 269.053 | 251 | 5.92 | 270.053 | [M-H]- | C10023 | "Baicalein (5,6,7-Trihydroxyflavone)" | Flavone |
| pme1514 | 305 | 125 | 2.72 | 306 | [M-H]- | C12136 | Epigallocatechin (EGC) | Flavone |
| pme1518 | 403.132 | 373 | 6.99 | 402.132 | [M+H]+ | C10112 | Nobiletin | Flavone |
| pme1521 | 303.058 | 125 | 4.07 | 304.058 | [M-H]- | C01617 | Dihydroquercetin (Taxifolin) | Flavone |
| pme1535 | 305 | 125 | 2.35 | 306.074 | [M-H]- | C12127 | (+)-Gallocatechin (GC) | Flavone |
| pme1539 | 623.169 | 314 | 3.74 | 624.169 | [M-H]- | - | Isorhamnetin 3-O-neohesperidoside | Flavone |
| pme1541 | 283.069 | 268 | 6.9 | 284.069 | [M-H]- | C01470 | Acacetin | Flavone |
| pme1550 | 373.121 | 343 | 7.47 | 372.121 | [M+H]+ | C10190 | Tangeretin | Flavone |
| pme1552 | 465.096 | 319 | 3.74 | 464.096 | [M+H]+ | C10108 | Myricetin 3-O-rhamnoside (Myricitrin) | Flavone |
| pme1562 | 441.3 | 169 | 3.81 | 442.3 | [M-H]- | - | Epicatechin gallate (ECG) | Flavonoid |
| pme1568 | 285.048 | 257 | 4.96 | 286.048 | [M-H]- | C10510 | "Orobol (5,7,3',4'-tetrahydroxyisoflavone)" | Flavonoid |
| pme1580 | 287.063 | 135 | 5 | 288.063 | [M-H]- | C05631 | Eriodictyol | Flavonoid |
| pme1588 | 315.058 | 151 | 5.78 | 316.058 | [M-H]- | C10084 | Isorhamnetin | Flavonoid |
| pme1598 | 463.132 | 301 | 3.99 | 464.132 | [M-H]- | - | Hesperetin 5-O-glucoside | Flavonoid |
| pme1599 | 301.079 | 165 | 6.22 | 302.079 | [M-H]- | - | 7-O-Methyleriodictyol | Flavonoid |
| pme1601 | 417.111 | 271 | 4.86 | 416.111 | [M+H]+ | - | Apigenin 4-O-rhamnoside | Flavonoid |
| pme1605 | 593.159 | 285 | 3.8 | 594.159 | [M-H]- | - | Kaempferol 3-O-robinobioside (Biorobin) | Flavonoid |
| pme1611 | 433.121 | 313 | 3.72 | 434.121 | [M-H]- | - | Isohemiphloin | Flavonoid |
| pme1624 | 433.106 | 283 | 3.7 | 432.106 | [M+H]+ | C01714 | Isovitexin | Flavonoid |
| pme1662 | 287.084 | 167 | 6.95 | 286.084 | [M+H]+ | C09833 | sakuranetin | Flavonoid |
| pme1667 | 595.2 | 415 | 3.3 | 594.159 | [M+H]+ | C08064 | Isovitexin 7-O-glucoside (Saponarin) | Flavonoid |
| pme1773 | 595 | 287 | 2.58 | 595 | Protonated | C08620 | Cyanidin 3-O-rutinoside (Keracyanin) | Flavonoid |
| pme1777 | 611 | 287 | 2.08 | 611 | Protonated | C08639 | "Cyanidin 3,5-O-diglucoside (Cyanin)" | Flavonoid |
| pme1793 | 595 | 270.9 | 2.42 | 595 | Protonated | C08725 | Pelargonin | Flavonoid |
| pme1824 | 153.1 | 109 | 2.5 | 154.027 | [M-H]- | C00230 | Protocatechuic acid | Flavonoid |
| pme2247 | 303 | 285 | 6.83 | 302.006 | [M+H]+ | C10788 | Ellagic acid | Flavonoid |
| pme2319 | 301.079 | 164 | 5.78 | 302.079 | [M-H]- | C01709 | Hesperetin | Flavonoid |
| pme2457 | 447.3 | 285.2 | 3.79 | 448.101 | [M-H]- | - | Luteolin 7-O-glucoside (Cynaroside) | Flavonoid |
| pme2478 | 137.1 | 108.4 | 3.04 | 138.032 | [M-H]- | C16700 | Protocatechuic aldehyde | Flavonoid |
| pme2493 | 579.2 | 287.1 | 3.74 | 578.164 | [M+H]+ | C16981 | "Kaempferol 3,7-dirhamnoside (Kaempferitrin)" | Flavonoid |
| pme2898 | 321.05 | 303 | 3.48 | 320.053 | [M+H]+ | C02906 | Dihydromyricetin | Flavonoid |
| pme2949 | 609.19 | 301 | 4.11 | 610.19 | [M-H]- | C09755 | Hesperetin 7-rutinoside (Hesperidin) | Flavonoid |
| pme2954 | 303.043 | 153 | 5.05 | 302.043 | [M+H]+ | C00389 | Quercetin | Flavonoid |
| pme2957 | 271.07 | 151 | 5.56 | 272.069 | [M-H]- | C06561 | Naringenin chalcone | Flavonoid |
| pme2963 | 287.06 | 259 | 4.56 | 288.063 | [M-H]- | C00974 | Aromadedrin (Dihydrokaempferol) | Flavonoid |
| pme2982 | 257.1 | 153 | 6.98 | 256.074 | [M+H]+ | C09827 | Pinocembrin (Dihydrochrysin) | Flavonol |
| pme2984 | 593 | 285 | 4.91 | 594.195 | [M-H]- | C09830 | Isosakuranetin-7-neohesperidoside (Poncirin) | Flavonol |
| pme3130 | 465 | 303 | 4.14 | 464.096 | [M+H]+ | - | Quercetin 4'-O-glucoside (Spiraeoside) | Flavonol |
| pme3208 | 445 | 283 | 3.51 | 446.121 | [M-H]- | C16195 | Glycitin | Flavonol |
| pme3211 | 463 | 301 | 3.78 | 464.096 | [M-H]- | C05623 | Quercetin 3-O-glucoside (Isotrifoliin) | Flavonol |
| pme3215 | 255 | 135 | 6.02 | 256.074 | [M-H]- | C08650 | Isoliquiritigenin | Flavonol |
| pme3268 | 449 | 287 | 3.96 | 448.101 | [M+H]+ | C12626 | Kaempferol 3-O-galactoside (Trifolin) | Flavonol |
| pme3276 | 285 | 217 | 4.91 | 286.048 | [M-H]- | C12134 | 2'-Hydroxygenistein | Flavonol |
| pme3285 | 275 | 139 | 3.4 | 274.084 | [M+H]+ | C09320 | "Afzelechin (3,5,7,4'-Tetrahydroxyflavan)" | Flavonol |
| pme3288 | 329 | 314 | 6.57 | 330.074 | [M-H]- | C01265 | "3,7-Di-O-methylquercetin" | Flavonol |
| pme3292 | 283 | 268 | 7.09 | 284.069 | [M-H]- | C10521 | Prunetin | Flavonol |
| pme3296 | 431 | 285 | 4.4 | 432.106 | [M-H]- | C16911 | Kaempferol 3-O-rhamnoside (Kaempferin) | Flavonol |
| pme3303 | 303 | 257 | 4.48 | 302.043 | [M+H]+ | C10192 | Tricetin | Flavonol |
| pme3369 | 315 | 165 | 6.31 | 316.058 | [M-H]- | C10176 | Rhamnetin (7-O-methxyl quercetin) | Flavonol |
| pme3391 | 479 | 317 | 2.61 | 479 | Protonated | C12139 | Petunidin 3-O-glucoside | Flavonol |
| pme3392 | 433.1 | 271 | 2.75 | 433.1 | Protonated | - | Pelargonidin 3-O-beta-D-glucoside（Callistephin chloride) | Flavonol |
| pme3393 | 287 | 149 | 3.78 | 288.063 | [M-H]- | C01378 | Fustin | Flavonol |
| pme3399 | 445.12 | 283 | 5.18 | 446.121 | [M-H]- | C05376 | Sissotrin | Flavonol |
| pme3401 | 345 | 330 | 5.71 | 346.069 | [M-H]- | C11620 | Syringetin | Flavonol |
| pme3407 | 331 | 151 | 5.07 | 332.053 | [M-H]- | C12633 | Laricitrin | Flavonol |
| pme3442 | 477.1 | 301 | 3.65 | 478.36 | [M-H]- | - | Quercetin 7-O-β-D-Glucuronide | Flavonol |
| pme3461 | 301.1 | 151 | 5.68 | 302.27876 | [M-H]- | C09756 | Homoeriodictyol | Flavonol |
| pme3464 | 285.08 | 164 | 6.92 | 286.084 | [M-H]- | C05334 | Isosakuranetin (4'-Methylnaringenin) | Flavonol |
| pme3473 | 271.07 | 151 | 5.56 | 272.069 | [M-H]- | C09614 | Butin | Flavonol |
| pme3484 | 479.09 | 315.5 | 3.43 | 480.09 | [M-H]- | - | Myricetin 3-O-galactoside | Flavonol |
| pme3502 | 429.13 | 267 | 4.57 | 430.126 | [M-H]- | C10509 | Formononetin 7-O-glucoside (Ononin) | Flavonol |
| pme3509 | 255.06 | 136.8 | 4.66 | 254.058 | [M+H]+ | C12123 | "7,4'-Dihydroxyflavone" | Flavonol |
| pme3514 | 301 | 151 | 5.05 | 302.04265 | [M-H]- | C10105 | Morin | Flavonol |
| pme3609 | 287 | 213 | 3.37 | 287.24 | Protonated | C05905 | Cyanidin | Flavonol |
| pmf0011 | 593.2 | 473 | 3.08 | 594.1585 | [M-H]- | - | "Apigenin 6,8-C-diglucoside" | Flavonol |
| pmf0012 | 595.2 | 457.2 | 3.08 | 594.1585 | [M+H]+ | - | "6,8-di-C-glucoside Apigenine" | Flavonol |
| pmf0027 | 449.1 | 286.8 | 2.56 | 448.1006 | [M]+ | C08647 | Cyanidin 3-O-galactoside | Flavonol |
| pmf0057 | 271.07 | 151 | 5.59 | 272.0685 | [M-H]- | C06561 | "4,2',4',6'-Tetrahydroxychalcone " | Flavonol |
| pmf0058 | 271 | 150.894 | 5.59 | 272.0685 | [M-H]- | C00509 | "4',5,7-Trihydroxyflavanone" | Flavonol |
| pmf0109 | 312.9 | 253 | 7.09 | 314.0790382 | [M-H]- | - | 3-O-Acetylpinobanksin | Flavonol |
| pmf0115 | 269 | 168.6 | 7.15 | 270.0528234 | [M-H]- | C10044 | Galangin | Flavonol |
| pmf0179 | 623.169 | 315.1 | 3.91 | 624.169 | [M-H]- | - | Narcissoside | Flavonol |
| pmf0204 | 465.0955 | 303 | 3.79 | 464.0955 | [M+H]+ | - | Hyperoside | Flavonol |
| pmf0208 | 465.0955 | 303 | 3.79 | 464.0955 | [M+H]+ | - | Isoquercitroside | Flavonol |
| pmf0216 | 443.09 | 272.8 | 3.84 | 442.09 | [M+H]+ | - | Catechin gallate，CG | Isoflavone |
| pmf0233 | 595.1373409 | 291 | 4.83 | 594.1373409 | [M+H]+ | C17140 | Tiliroside | Isoflavone |
| pmf0238 | 715.137756 | 563.3 | 4.71 | 716.137756 | [M-H]- | - | Theaflavin-3-gallate | Isoflavone |
| pmf0240 | 715.1377348 | 563.3 | 4.8 | 716.1377348 | [M-H]- | - | Theaflavin | Isoflavone |
| pmf0246 | 449.1005615 | 327 | 3.49 | 448.1005615 | [M-H]- | C10114 | Orientin | Isoflavone |
| pmf0260 | 373.120903 | 312.8 | 6.52 | 372.120903 | [M+H]+ | C10186 | Sinensetin | Isoflavone |
| pmf0265 | 177.0266087 | 88.9 | 4.41 | 178.0266087 | [M-H]- | C09001 | "5,7-Dihydroxychromone" | Isoflavone |
| pmf0274 | 301.042664 | 211.4 | 5.23 | 302.042664 | [M-H]- | - | Herbacetin | Polyphenol |
| pmf0279 | 465.09549 | 229.2 | 3.74 | 464.09549 | [M+H]+ | - | Gossypitrin | Polyphenol |
| pmf0302 | 435.1213 | 289.1 | 4.22 | 434.1213 | [M+H]+ | - | Engeletin | Polyphenol |
| pmf0330 | 607.2 | 445 | 3.77 | 608.1741 | [M-H]- | C17834 | Spinosin | Polyphenol |
| pmf0344 | 273.1 | 189 | 3.65 | 274.0841 | [M-H]- | C12128 | (-)-Epiafzelechin | Polyphenol |
| pmf0361 | 451.1 | 71 | 3.92 | 450.11621 | [M+H]+ | C17449 | Astilbin | Polyphenol |
| pmf0362 | 299.1 | 227 | 6.29 | 300.06339 | [M-H]- | - | Hydroxygenkwanin | Polyphenol |
| pmf0369 | 477.1 | 315 | 4.49 | 478.14751 | [M-H]- | - | Persicoside | Polyphenol |
| pmf0371 | 315.050489 | 300 | 4.65 | 316.058289 | [M-H]- | C10119 | Pedalitin | Polyphenol |
| pmf0374 | 477.103315 | 314 | 4.1 | 478.111115 | [M-H]- | - | Isorhamnetin 3-O-glucoside | Polyphenol |
| pmf0379 | 457.0771 | 169 | 3.37 | 458.0849 | [M-H]- | - | Gallocatechin gallate | Polyphenol |
| pmf0382 | 343.0818 | 313 | 6.74 | 344.0896 | [M-H]- | C19807 | "5,7-Dihydroxy-3',4',5'-trimethoxyflavone" | Polyphenol |
| pmf0392 | 315.0869 | 178 | 7.09 | 316.0947 | [M-H]- | - | Persicogenin | Polyphenol |
| pmf0458 | 295.2 | 277 | 6.97 | 294.18311 | [M+H]+ | C10462 | 6-Gingerol | Polyphenol |
| pmf0472 | 565.1557 | 271.1 | 3.97 | 564.1479 | [M+H]+ | C04858 | Apiin | Polyphenol |
| pmf0550 | 609.1818 | 301 | 4.05 | 608.174 | [M+H]+ | C10039 | Diosmin | Polyphenol |
| pmf0567 | 299.1 | 211 | 5.69 | 300.063385 | [M-H]- | C10534 | Tectorigenin | Polyphenol |
| pmf0582 | 325.1 | 123 | 7.91 | 324.136169 | [M+H]+ | C10421 | Glabridin | Polyphenol |
| pmf0583 | 435.1 | 167 | 4.33 | 436.136932 | [M-H]- | C01604 | Phloridzin | Polyphenol |
| pmf0615 | 641.4 | 317 | 2.28 | 641.4 | Protonated | - | "Petunidin 3, 5-diglucoside" | Proanthocyanidins |
| pmf0616 | 697.1 | 535.1 | 3 | 697.1 | Protonated | - | Malvidin 3-acetyl-5-diglucoside | Proanthocyanidins |
| pmf0617 | 787.23 | 463.4 | 2.39 | 787.23 | Protonated | - | Peonidin 3-sophoroside-5-glucoside. | Proanthocyanidins |

Note: Parent ion value (Q1) ,characteristic fragment ions value (Q3),retention time(RT)
