# Supplementary material for: Comprehensive analysis of metabolome and transcriptome reveals the mechanism of color formation in different leave of Loropetalum Chinense var. Rubrum
Source: BMC Plant Biol. 2023 Mar 8;23:133. doi: 10.1186/s12870-023-04143-9 (PMC9993627; doi:10.1186/s12870-023-04143-9)
Supplement: Supplementary file 14 — Additional file 14: Fig. S3. Correlation analysis of transcription factors and structural genes [file 12870_2023_4143_MOESM14_ESM.docx]

**Additional files 5:Table S4.**

Table S5. Statistical analysis of *L. chinense var. rubrum* reads in 9 libraries (Each sample was repeated three times)

| **Library ID** | **Sample name** | **Raw reads** | **Clean reads** | **Clean bases** | **Error rate(%)** | **Q20(%)** | **Q30(%)** | **GC content(%)** |
| --- | --- | --- | --- | --- | --- | --- | --- | --- |
| FRAS190049924-1a | GL1 | 82,265,722 | 81,385,416 | 12.21G | 0.02 | 98.11 | 94.33 | 43.72 |
| FRAS190049925-1a | GL2 | 45,504,946 | 44,090,072 | 6.61G | 0.02 | 98.10 | 94.41 | 43.56 |
| FRAS190049926-1a | GL3 | 58,923,902 | 58,157,216 | 8.72G | 0.02 | 98.06 | 94.34 | 43.90 |
| FRAS190049927-1a | PL1 | 49,725,148 | 49,208,224 | 7.38G | 0.02 | 98.17 | 94.51 | 43.89 |
| FRAS190049928-1a | PL2 | 46,297,074 | 45,802,144 | 6.87G | 0.03 | 97.52 | 92.88 | 44.00 |
| FRAS190049929-1a | PL3 | 53,924,164 | 52,538,740 | 7.88G | 0.02 | 98.01 | 94.31 | 43.64 |
| FRAS190049930-1a | ML1 | 41,183,014 | 40,613,790 | 6.09G | 0.03 | 97.49 | 92.88 | 44.24 |
| FRAS190049931-1a | ML2 | 41,319,012 | 40,856,576 | 6.13G | 0.03 | 97.47 | 92.82 | 44.01 |
| FRAS190049932-1a | ML3 | 40,641,036 | 40,168,464 | 6.03G | 0.03 | 97.48 | 92.87 | 43.69 |

Note:If the quality value is Q20, the probability of error rate recognition is 1%, or the correct rate is 99%;If the quality value is Q30, the error rate recognition probability is 0.1%, or the correct rate is 99.9%.
