# Supplementary material for: Comprehensive analysis of metabolome and transcriptome reveals the mechanism of color formation in different leave of Loropetalum Chinense var. Rubrum
Source: BMC Plant Biol. 2023 Mar 8;23:133. doi: 10.1186/s12870-023-04143-9 (PMC9993627; doi:10.1186/s12870-023-04143-9)
Supplement: Supplementary file 15 — Additional file 15: Table S12. List of primers used in this study [file 12870_2023_4143_MOESM15_ESM.docx]

**Additional files 9: Fig. S2.**


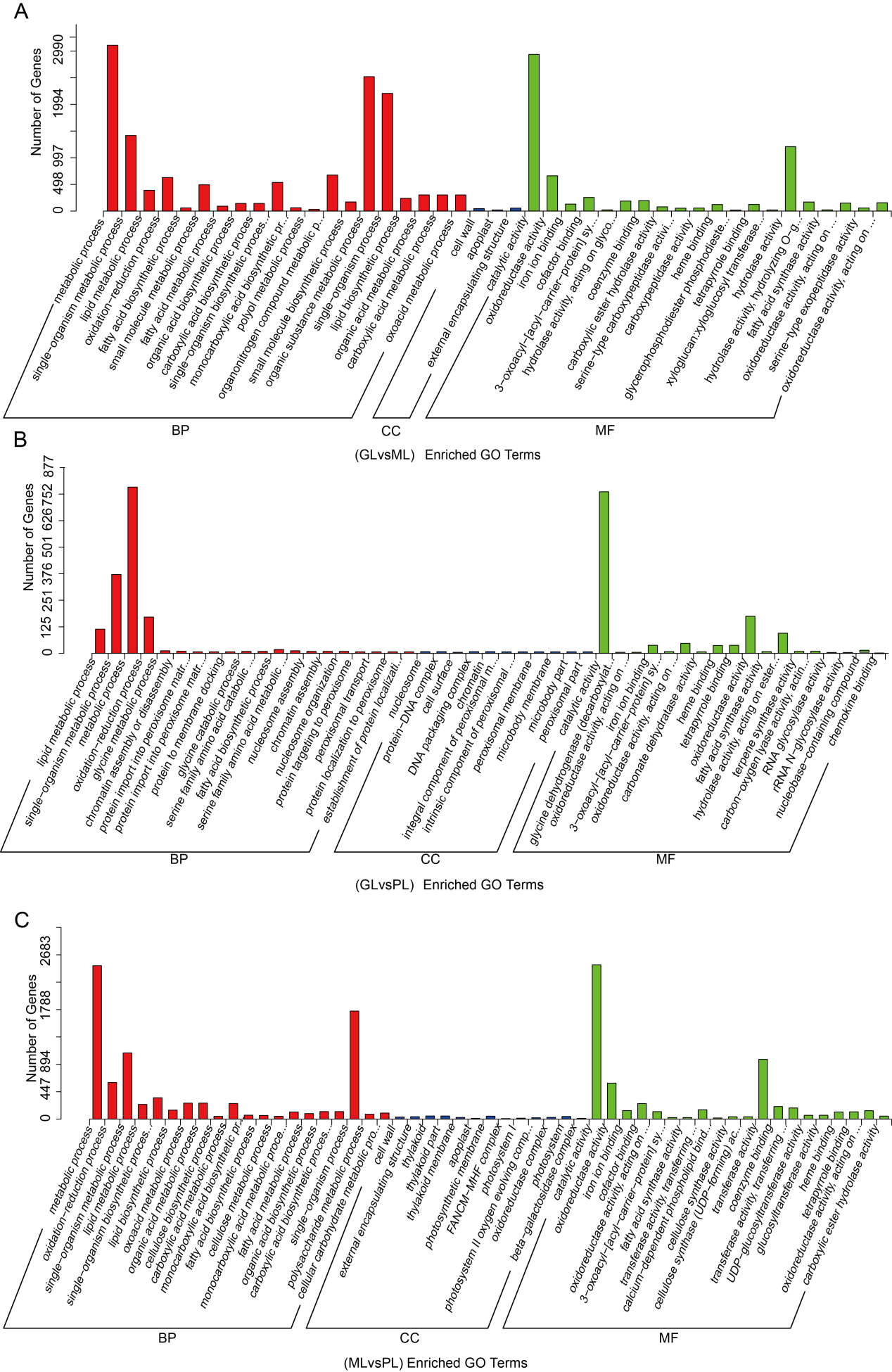


**Fig. S2.** GO classification of DEGs. **A** GO functional classification of DEGs between GL vs ML. **B** GO functional classification of DEGs between GL vs PL. **C** GO functional classification of DEGs between ML vs PL
